# The role of white matter microstructure in the association between childhood maltreatment and adult post-traumatic symptoms

**DOI:** 10.1038/s41380-026-03624-1

**Published:** 2026-10-10

**Authors:** Luisa Berger, Philine Rojczyk, Johanna Seitz-Holland, Fan Zhang, Lara Pankatz, Nico Sollmann, Elisabeth Kaufmann, Holly Carrington, Twishi Puri, Michael J. Coleman, Ofer Pasternak, Sylvain Bouix, Yogesh Rathi, Suheyla Cetin-Karayumak, Lauren J. O’Donnell, Peter Falkai, Mark S. George, Thomas W. McAllister, Ross Zafonte, Murray B. Stein, Martha E. Shenton, Inga K. Koerte

**Affiliations:** 1 Psychiatry Neuroimaging Laboratory, Department of Psychiatry, Brigham and Women’s Hospital, Harvard Medical School, Somerville, MA USA; 2 cBRAIN, Department of Child and Adolescent Psychiatry, Psychosomatics and Psychotherapy, LMU University Hospital, LMU Munich, Munich, Germany; 3 Department of Psychiatry, Massachusetts General Hospital, Harvard Medical School, Boston, MA USA; 4 Department of Radiology, Brigham and Women’s Hospital, Harvard Medical School, Boston, MA USA; 5 University of Electronic Science and Technology of China, Chengdu, Sichuan China; 6 Department of Diagnostic and Interventional Radiology, University Hospital Ulm, Ulm, Germany; 7 Department of Diagnostic and Interventional Neuroradiology, School of Medicine, Klinikum rechts der Isar, Technical University of Munich, Munich, Germany; 8 TUM-Neuroimaging Center, Klinikum rechts der Isar, Technical University of Munich, Munich, Germany; 9 Department of Neurology, LMU University Hospital, LMU Munich, Munich, Germany; 10 Brain Injury Research Center of Mount Sinai, Icahn School of Medicine at Mount Sinai, New York, USA; 11 Department of Software Engineering and IT, École de Technologie Supérieure, Montreal, Quebec, Canada; 12 Department of Psychiatry and Psychotherapy, University Hospital, LMU Munich, Munich, Germany; 13 Max Planck Institute of Psychiatry, Munich, Germany; 14 Psychiatry Department, Medical University of South Carolina, Charleston, USA; 15 Ralph H. Johnson VA Medical Center, Charleston, USA; 16 Department of Psychiatry, Indiana University School of Medicine, Indianapolis, USA; 17 Department of Physical Medicine and Rehabilitation, Spaulding Rehabilitation Hospital, Harvard Medical School, Charlestown, USA; 18 Department of Physical Medicine and Rehabilitation, Brigham and Women’s Hospital, Harvard Medical School, Boston, USA; 19 Department of Psychiatry, University of California San Diego, La Jolla, USA; 20 School of Public Health, University of California San Diego, La Jolla, USA; 21 Psychiatry Service, VA San Diego Healthcare System, San Diego, USA; 22 German Center for Child and Adolescent Health (DZKJ), partner site Munich, Munich, Germany

**Keywords:** Psychiatric disorders, Neuroscience, Psychology

## Abstract

Childhood maltreatment (CM) is a risk factor for adult post-traumatic symptoms and white matter (WM) alterations. It remains unknown how WM microstructure is affected alongside two common military conditions, post-traumatic stress disorder (PTSD) and mild traumatic brain injury (mTBI). Data from 299 participants (53% male, 35.69 ± 12.23 years of age) of the Injury and Traumatic Stress (INTRuST) clinical consortium was analyzed. The study included 56 individuals with PTSD (41 with comorbid mTBI), 107 with mTBI, and 136 controls (no PTSD and/or mTBI). Diffusion parameters (mean, axial, and radial diffusivity, MD, AD, RD; fractional anisotropy, FA) in major WM tracts were extracted from diffusion MRI. CM was assessed using the Childhood Trauma Questionnaire (CTQ). Multiple regression models examined associations between CM severity and WM diffusion. Moderation analyses assessed the effect of PTSD and/or mTBI on these associations. Mediation analyses examined how WM diffusion affects the associations between CM and both PTSD and post-concussive symptom severity. Greater CM severity was associated with lower MD and AD, independent of PTSD and/or mTBI. WM diffusion, particularly in the corpus callosum and the superior longitudinal fasciculus, mediated the associations between CM severity and both PTSD (MD b_ab_ = 0.022, AD b_ab_ = 0.019-0.034) and post-concussive symptoms (MD b_ab_ = 0.028-0.040, AD b_ab_ = 0.032-0.047). Findings suggest that WM alterations are more strongly associated with CM itself than with either PTSD and/or mTBI. Mediating effects in regions critical for sensory integration highlight the role of WM microstructure as a neurobiological pathway through which CM might predispose to post-traumatic symptoms.

## Introduction

Childhood maltreatment (CM) is widely recognized as a major risk factor for adult mental health problems such as depression, anxiety, substance use disorder, and post-traumatic stress disorder [1]. Notably, military service members report higher CM experience rates, with military personnel being twice as likely to report having experienced physical or sexual abuse in their childhood [2]. While the relationship between CM and adult mental health problems seems evident, the underlying pathophysiological mechanisms driving this connection remain largely unknown. Understanding these neural pathways is crucial for developing targeted interventions and preventive measures. This is particularly important for the vulnerable population of military personnel who may also be exposed to additional trauma from combat and war zone-related experiences [3, 4].

Although limited in number, neuroimaging studies consistently report that CM is associated with alterations in brain function, structure, volume, and structural connectivity in adulthood [5, 6]. Specifically, preliminary evidence suggests that CM may induce lasting structural alterations of the brain, thereby contributing to the susceptibility to further trauma exposure and developing psychopathology in adulthood [7]. Microstructural tissue alterations have been identified in individuals with history of CM in multiple white matter (WM) tracts such as the corpus callosum (CC) [8, 9], cingulum bundle [10], superior longitudinal fasciculus (SLF) [11, 12], and inferior longitudinal fasciculus (ILF) [13]. Olson et al. showed that ILF alterations associated with CM were evident even when accounting for current PTSD symptoms, underscoring the potential specificity of CM effects. These major WM pathways are primarily involved in the governance of cognitive and emotional functioning [14, 15], suggesting they may play a contributory role in the functional difficulties observed in these areas in individuals with a history of CM.

Considering the high rates of post-traumatic stress disorder (PTSD) and mild traumatic brain injury (mTBI) among military personnel [16], it is crucial to investigate the impact of CM on WM microstructure in the context of these “signature wounds” of military service. There are heterogeneous findings regarding the association of WM microstructure with PTSD [17, 18] and mTBI [19, 20]. Notably, WM alterations seem to be more pronounced in individuals with comorbid PTSD and mTBI compared to either condition alone, reflected in less organized tissue architecture [21, 22]. However, it remains unknown how history of CM relates to these observed associations.

This study aims to further our understanding of the relationships between CM severity, WM microstructure, and post-traumatic symptoms in the context of the common military conditions PTSD and mTBI. There are three main objectives: (1) we investigate to what extent CM severity is associated with WM microstructure in adults; (2) we examine the effect of PTSD and mTBI on the associations between CM severity and WM microstructure in adults; and (3) we explore potential mediating effects of WM microstructure on the associations between CM severity and PTSD and post-concussive symptoms.

## Materials and methods

### Study design and participants

Participants were enrolled between 2008 and 2013 as part of the Department of Defense Injury and Traumatic Stress (INTRuST) clinical consortium (Department of Defense, W81XWH-08-2-0159) at ten sites across the United States, aiming to enhance understanding and treatment of PTSD and mTBI in military service personnel [21, 23–29]. The study was approved by the Institutional Review Board, Brigham and Women’s Hospital (2010P002516), and conducted following the Declaration of Helsinki. Written informed consent was obtained from all study participants prior to enrollment.

To be included in the INTRuST study, participants had to be English-speaking males or females between the ages of 18 and 75 years. Participants were excluded if they had (1) acquired English as a second language after the age of 5 years, (2) a history of a learning disability, (3) a history of moderate or severe TBI, (4) a diagnosis of bipolar I disorder, (5) psychosis delirium, or dementia-related disorders, (6) uncontrolled hypertension or chronic disease, (7) were taking more than one antihypertensive medication, (8) were taking oral or intramuscular steroids within the last four months, or (9) were currently taking medication affecting brain function (other than psychotropic medications). Due to the high prevalence of psychoactive medication and substance abuse in this study sample, a history of psychotropic drug, alcohol, or substance use was permitted if in remission for the last 30 days prior to enrollment. Other exclusion criteria were (1) MRI contraindications, (2) disorders of the central nervous system, or (3) pregnancy or lactation.

Out of the 771 INTRuST study participants, 335 were excluded due to missing MRI data, 44 due to insufficient MRI data quality, and 39 due to data harmonization issues related to insufficient participant numbers at certain sites. In the present study, we included participants who had both MRI data and a complete set of assessments of CM, PTSD, and mTBI, yielding a final sample of 299 participants (Figure S1).

### Diagnostic and clinical assessments

#### Assessment of severity of childhood maltreatment

The Childhood Trauma Questionnaire (CTQ) is a 28-item self-report questionnaire applied in adults to assess a history of maltreatment experienced during childhood [30, 31]. The CTQ evaluates five types of traumatic childhood experiences: emotional abuse, emotional neglect, physical abuse, physical neglect, and sexual abuse. Individuals rate each item on a Likert scale from *1* (“never true”) to *5* (“very often true”), indicating the severity of CM. Subscale scores were obtained by summing the item scores of each corresponding subscale. A total CM score was computed by summing items across all subscales.

In this study, CTQ data were available as subscale totals. Item-level responses were not provided, precluding calculation of the Minimization/Denial validity scale. Subscale totals were range-checked for plausibility and categorized using established severity cutoffs (Supplementary Analyses, Tables S9 and S11).

#### Assessment of PTSD

Multiple assessment tools were utilized to diagnose PTSD, including the Mini-International Post-traumatic Interview (MINI) [32], the PTSD Checklist [33], the Clinician-Administered PTSD Scale for Diagnostic and Statistical Manual of Mental Disorders (DSM)-5 (CAPS) [34], and the Structured Clinical Interview for DSM-4 (SCID) [35].

Current severity and presence of PTSD symptoms were assessed using the PTSD Checklist-Civilian Version (PCL-C) [33]. This 17-item questionnaire aligns with the diagnostic criteria of the DSM-4 [36]. Items are rated on a scale from *1* (“not at all bothersome”) to *5* (“extremely bothersome”) and summed into a total PTSD symptom severity score.

In addition, depressive symptom severity was evaluated using the Patient Health Questionnaire-9 (PHQ-9) [37], a validated self-report measure, by summing the scores of all nine items.

#### Assessment of mTBI and post-concussive symptoms

History of mTBI was assessed using the INTRuST mTBI screening instrument, which follows the diagnostic guidelines of the American Congress of Rehabilitation Medicine [38]. This self-report questionnaire comprises three items that evaluate: (1) prior head injuries, (2) immediate loss or alteration of consciousness or unawareness of the event, and (3) amnesia preceding or following the incident. Post-concussive symptoms were evaluated using the Rivermead Post-Concussion Symptoms Questionnaire (RPQ13) [39]. The RPQ-13 is designed to measure chronic and persistent post-concussive symptoms, demonstrating robust internal validity, test-retest reliability, and inter-rater reliability [40]. A higher score on the RPQ13 indicates a higher severity of post-concussive symptoms.

### MRI acquisition and image processing

#### Image acquisition

Diffusion-weighted MRI (dMRI) was obtained using 3-Tesla scanners (Tim Trio, Siemens Healthineers, Erlangen, Germany; GE 750, GE Healthcare, Chicago, IL, USA; Achieva, Philips Healthcare, Best, The Netherlands) at six out of the ten INTRuST study sites. The dMRI acquisition parameters on the different systems are detailed in Table S1.

#### Pre-processing and image harmonization

The dMRI data were pre-processed using our in-house pipeline (https://github.com/pnlbwh/pnlNipype), applying axis alignment, centering, motion correction, and eddy current correction [41, 42]. Image quality was assessed visually and semi-automatically using 3D Slicer (version 4.5, http://www.slicer.org) [43]. Diffusion masks were generated and manually corrected as required using 3D Slicer. To address the multi-site nature of the INTRuST study and to account for scanner variability, we used a single-shell harmonization algorithm (https://github.com/pnlbwh/dMRIharmonization) on the dMRI data (b = 900 s/mm^2^) [44, 45]. This mitigated scanner-specific differences while preserving the within-site inter-subject variability [46].

#### White matter fiber clustering

Fiber clustering for WM was performed using the open-source whitematteranalysis (WMA) software pipeline (https://github.com/SlicerDMRI/whitematteranalysis; Figure S2). This method groups fibers based on anatomical shape and spatial position, extracting numerous fiber tracts covering the entire brain [47]. The WM fiber clustering generates the WM parcellation of each individual subject based on a pre-provided fiber clustering atlas, which was curated by a neuroanatomist (http://dmri.slicer.org/atlases/).

Initially, two-tensor whole-brain unscented Kalman Filter (UKF) tractography (https://github.com/pnlbwh/ukftractography) was employed [48]. We used the following parameters: --Ql 70, --Qm 0.001, --Rs 0.015, --numTensor 2, --seedFALimit 0.18, --seedsPerVoxel 10, --stepLength 0.2. Prior research has shown that the UKF method exhibits high consistency [47] and greater sensitivity compared to single-tensor tractography [49–51]. The two-tensor model was selected to account for crossing fibers [52, 53], with the first tensor representing the primary direction of the fiber tract and the second tensor accounting for crossing fibers. Anatomical accuracy and alignment with the atlas tractography data were ensured by using the WMA software quality control tool [54].

Subsequently, we employed the WMA software for tract parcellation to identify WM tracts for each subject. For this purpose, the tractography of each subject was registered to the pre-provided atlas clusters corresponding to each WM tract [47, 55, 56]. This minimized the occurrence of false-positive tractography results, thus enhancing the overall reliability of tract parcellation [54].

**Fig. 1:**
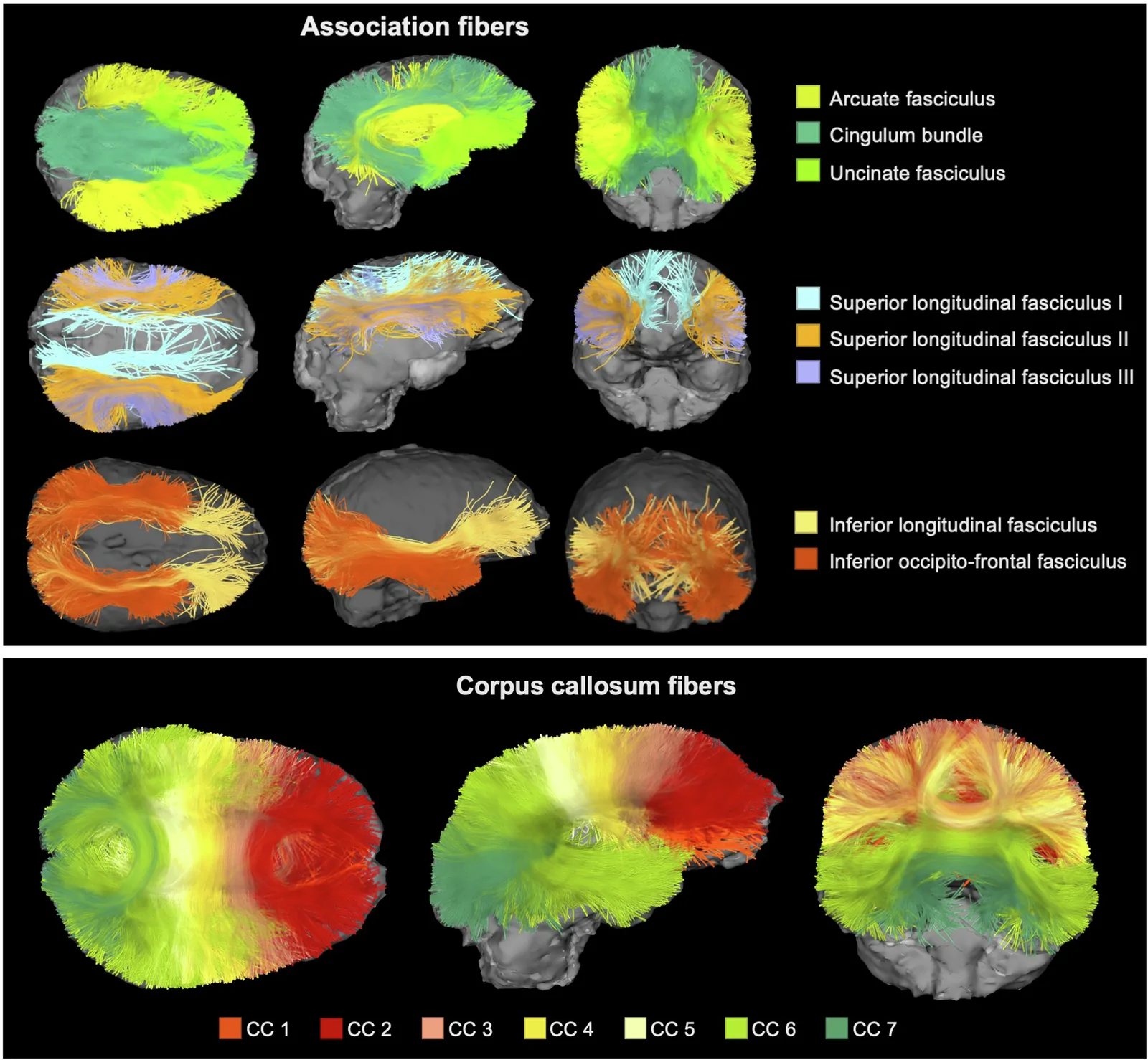
White matter tracts included in the analyses. Association fibers and CC fibers were included in our analyses. WM tracts consist of grouped fiber clusters. Predefined subregions of the CC (CC1–CC7) [59] were selected, and WM fibers passing through each subregion are depicted in different colors. *Note*. CC corpus callosum.

Major WM tracts previously associated with CM were extracted (left/right arcuate fasciculus [11], left/right cingulum bundle [10], CC [8, 9], left/right inferior longitudinal fasciculus [57], left/right inferior occipito-frontal fasciculus [10], left/right SLF [11, 12], and left/right uncinate fasciculus [58]). The CC was divided into seven anatomical subregions [59]: CC1=rostrum, CC2=genu, CC3 + 4=anterior half of the body, CC5 + 6=posterior half of the body, and CC7=splenium (Fig. 1**)**.

To ensure anatomical accuracy, we quantitatively assessed the number of fibers and visually inspected each subject’s WM tracts as previously described [60–63]. One case had to be excluded during the quality check process due to an insufficient number of fibers (CC5).

#### Diffusion parameter extraction

Diffusion parameters were extracted in the major WM tracts. Specifically, mean diffusivity (MD), axial diffusivity (AD), radial diffusivity (RD), and fractional anisotropy (FA) corrected for free-water [64] were extracted, which collectively reflect the extent and directionality of water diffusion [65–68]. In detail, MD represents the average magnitude of diffusion along all three spatial axes of the WM tract [69]. AD accounts for the magnitude of diffusion along the main axis of the WM tract, encompassing diffusion anisotropy components associated with axonal structure, directionality, and branching. RD measures the magnitude of diffusion perpendicular to the main axis of the WM tract [70–72]. FA is a summary measure of tissue organization [73]. We used free-water correction to alleviate the effects of partial volume with cortico-spinal fluid or extracellular free-water on the diffusivity measures [64]. Free-water correction provides a more accurate assessment of cellular WM structure compared to the conventional approach [74].

### Statistical analyses

All statistical analyses were conducted using R (version 4.3.1; R Core Team, Vienna, Austria) [75]. We created figures using R 4.3.1 and PowerPoint 16.75. To account for multiple testing, we applied the Benjamini-Hochberg method with a false-discovery rate (FDR) of 5% [76]. A p-value < 0.05 was considered statistically significant.

**Fig. 2:**
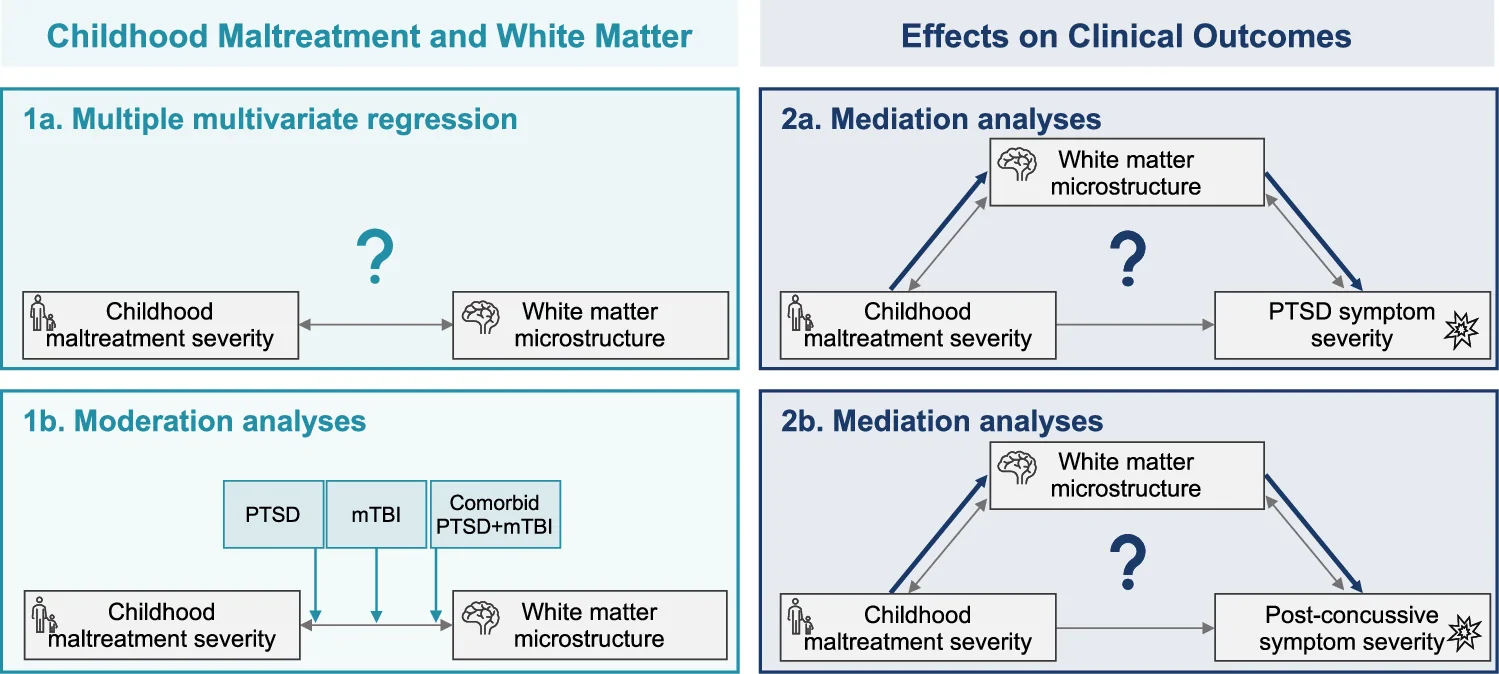
Hierarchical statistical approach. This figure illustrates the hierarchical statistical approach used in the study. The analysis is structured in three main levels. First, we examined the association between CM severity and WM microstructure (**1a**). Second, we investigated how PTSD and mTBI affect the relationship between CM severity and WM microstructure (**1b**). Third, we explored the potential mediating effects of WM microstructure on the associations between CM severity and PTSD symptom severity (**2a**) and post-concussive symptom severity (**2b**). *Note*. PTSD post-traumatic stress disorder, mTBI mild traumatic brain injury.

All models included age and gender as covariates. Post-hoc analyses additionally included race and alcohol abuse as covariates. Given the observed high multicollinearity (r = 0.85; *p* < 0.001) between PTSD symptom severity (PCL-C/PCL-M) and depressive symptom severity (PHQ-9), we refrained from including it as a covariate. Figure 2 summarizes the statistical approach.

#### Associations between childhood maltreatment severity and white matter microstructure

The association between total CM severity and WM microstructure was analyzed using multiple regression models between CM severity (CTQ total score) as the independent variable and dMRI measurements (MD, AD, RD, and FA) as the dependent variables. For each diffusion modality, we conducted 23 regression models, one for each pre-selected white matter tract. FDR correction was applied to account for the 23 comparisons within each diffusion modality. Additional analyses between the CM subscales (emotional abuse, emotional neglect, physical abuse, physical neglect, and sexual abuse) and WM microstructure are provided in the Supplementary Analyses. To assess robustness, additional analyses were conducted and are reported in the Supplementary Analyses.

#### Moderating effect of PTSD and/or mTBI on the association between childhood maltreatment severity and white matter microstructure

In case of a statistically significant association between CM severity and WM microstructure, we tested the moderating effect of PTSD, mTBI, and comorbid PTSD+mTBI on this association using the R PROCESS macro (version 4, model 2) [77]. Diagnoses were coded as binary indicators (1 = present, 0 = absent) and entered as moderators. Sensitivity and robustness checks, including tests of subgroup imbalance and comorbidity, are reported in the Supplementary Analyses.

#### Mediating effect of white matter microstructure on the association of childhood maltreatment severity with post-traumatic symptoms

Mediation analyses explored whether WM microstructure mediates the associations between CM severity and PTSD or post-concussive symptom severity. All mediation analyses were conducted using the R PROCESS macro (version 4, model 4) [77], which employs non-parametric bootstrapping with *N* = 5000 permutations to generate 95% bias-corrected confidence intervals. The dMRI measures that showed significant associations with CM severity were included as mediators. Two mediation models were utilized, with CM severity as the independent variable in both models, PCL-C/PCL-M scores as the dependent variable in the first model, and RPQ-13 scores as the dependent variable in the second model. Further examinations of the CM subscales and WM microstructure are detailed in the Supplementary Analyses.

## Results

Demographic and clinical characteristics of the included subjects are presented in Table 1. Overall, data from 299 participants (53% male, mean age = 35.69 ± 12.23 years) were analyzed, including 56 individuals with PTSD (41 with comorbid mTBI), 107 with mTBI, and 136 controls without any history of PTSD and/or mTBI.

**Table 1:** Demographic and clinical characteristics.

	N	n	Mean (SD); % of total sample	Range
**Demographic characteristics**				
**Age (years)**	299		36.51 (12.35)	18 – 65
**Gender**	299			
Male		168	56%	
Female		131	44%	
**Race**	288			
Native		2	0.7%	
Asian		9	3.1%	
Pacific		1	0.3%	
African American		41	14%	
White		231	80%	
Unknown		4	1.4%	
**Clinical characteristics**				
**CTQ**	299		37.67 (17.69)	25 – 115
**PCL-C/PCL-M**	299		29.46 (17.25)	17 – 85
**RPQ-13**	177		14.23 (14.95)	0 – 52
**PTSD diagnosis**				
PTSD		56	19%	
No PTSD		243	81%	
**mTBI diagnosis**				
mTBI		148	49%	
No mTBI		151	51%	
**PHQ-9**	299		4.07 (5.53)	0 – 27

*CTQ* childhood trauma questionnaire, *PCL-C* PTSD checklist civilian-version, *PCL-M* PTSD checklist military-version, *RPQ-13* rivermead post-concussion symptoms questionnaire, *PHQ-9* patient health questionnaire-9.

PTSD and mTBI diagnostic categories are not mutually exclusive; 41 participants were comorbid for PTSD and mTBI.

### Associations between childhood maltreatment severity and white matter microstructure

Higher CM severity was significantly associated with lower MD and lower AD of various WM tracts, including the right arcuate fasciculus, the genu and posterior body region of the CC, the ILF, the left inferior occipito-frontal fasciculus, and the right uncinate fasciculus (Table 2). Moreover, we found significant negative associations between CM severity and AD in the left arcuate fasciculus, the right cingulum bundle, the anterior and posterior body region of the CC, and the left and right SLF I, II, and III (Figure S3). Notably, no significant associations were observed between CM severity and FA, nor between CM severity and RD (Table 2). Subsequent adjustments for race and alcohol abuse did not change the significance for most associations (Table S2). Results were consistent across robustness checks and when excluding the PTSD-only subgroup (Tables S5-S6).

**Table 2:** Multiple regression results between childhood maltreatment severity and white matter microstructure.

	Fractional anisotropy (FA)		Mean diffusivity (MD)		Axial diffusivity (AD)		Radial diffusivity (RD)	
	p_FDR_	beta_._	p_FDR_	beta_._	p_FDR_	beta_._	p_FDR_	beta_._
AF left	0.549	−0.052	0.074	−0.122	**0.029**	−**0.134**	0.944	0.006
AF right	0.955	−0.005	**0.028**	−**0.152**	**0.035**	−**0.131**	0.587	−0.051
CB left	0.804	−0.020	0.493	−0.050	0.110	−0.102	0.833	−0.020
CB right	0.518	−0.059	0.183	−0.093	**0.009**	−**0.163**	0.642	−0.043
CC 1	0.953	0.006	**0.015**	−**0.167**	0.103	−0.107	0.555	−0.057
CC 2	0.975	−0.003	**0.007**	−**0.180**	**0.027**	−**0.139**	0.544	−0.060
CC 3	0.798	−0.021	0.072	−0.126	**0.001**	−**0.209**	0.649	−0.042
CC 4	0.953	−0.007	0.085	−0.118	**0.006**	−**0.170**	0.587	−0.052
CC 5	0.690	−0.036	0.061	−0.132	**0.006**	−**0.175**	0.762	−0.029
CC 6	0.591	−0.046	**0.003**	−**0.193**	**0.021**	−**0.149**	0.817	−0.023
CC 7	0.552	−0.052	0.064	−0.129	0.204	−0.085	0.975	−0.003
ILF left	0.716	0.033	0.274	−0.078	0.471	−0.050	0.587	−0.053
ILF right	0.780	0.024	**0.042**	−**0.144**	0.074	−0.115	0.345	−0.080
IoFF left	0.794	−0.022	**0.003**	−**0.196**	**0.024**	−**0.144**	0.616	−0.047
IoFF right	0.737	−0.030	**0.018**	−**0.161**	0.050	−0.123	0.783	−0.026
SLF I left	0.616	−0.044	0.077	−0.120	**0.007**	−**0.165**	0.610	−0.049
SLF I right	0.549	−0.054	0.211	−0.085	**0.022**	−**0.142**	0.844	−0.017
SLF II left	0.370	−0.075	0.298	−0.072	**0.024**	−**0.140**	0.769	0.028
SLF II right	0.359	−0.076	0.158	−0.100	**0.005**	−**0.170**	0.902	0.011
SLF III left	0.794	−0.022	0.178	−0.095	0.241	−0.077	0.902	−0.012
SLF III right	0.506	−0.061	0.141	−0.104	**0.030**	−**0.134**	0.844	0.019
UF left	0.549	−0.053	0.074	−0.124	**0.000**	−**0.275**	0.762	−0.030
UF right	0.780	0.025	**0.042**	−**0.144**	**0.001**	−**0.210**	0.363	−0.077

Higher childhood maltreatment severity was significantly associated with lower mean diffusivity and axial diffusivity in various white matter tracts. No significant associations were found with fractional anisotropy or radial diffusivity. All analyses were corrected for age and gender as covariates.

*FA* fractional anisotropy, *MD* mean diffusivity, *AD* axial diffusivity, *RD* radial diffusivity, *FDR* false-discovery rate, *AF* arcuate fasciculus, *CB* cingulum bundle, *CC* corpus callosum, *ILF* inferior longitudinal fasciculus, *IoFF* inferior occipito-frontal fasciculus, *SLF* superior longitudinal fasciculus, *UF* uncinate fasciculus. Bold values indicate statistical significance after FDR correction (p_FDR_ < 0.05).

### Moderating effect of PTSD and/or mTBI on the association between childhood maltreatment severity and white matter microstructure

The moderation analyses revealed that the significant associations between CM severity and WM microstructure persisted independent of a diagnosis of PTSD (all *p* > 0.05), mTBI (all *p* > 0.05), or comorbid PTSD+mTBI (all *p* > 0.05). Robustness checks confirmed that moderation effects were overall nonsignificant across tracts, including the three-way CM×PTSD×mTBI interaction (Tables S7-S8).

### Mediating effect of white matter microstructure on the association of childhood maltreatment severity with post-traumatic symptoms

Given the significant associations between CM severity and WM microstructure (Table 2), we performed additional mediation analyses to explore whether WM microstructure mediates the associations between CM severity and PTSD symptom severity and post-concussive symptom severity.

**Fig. 3:**
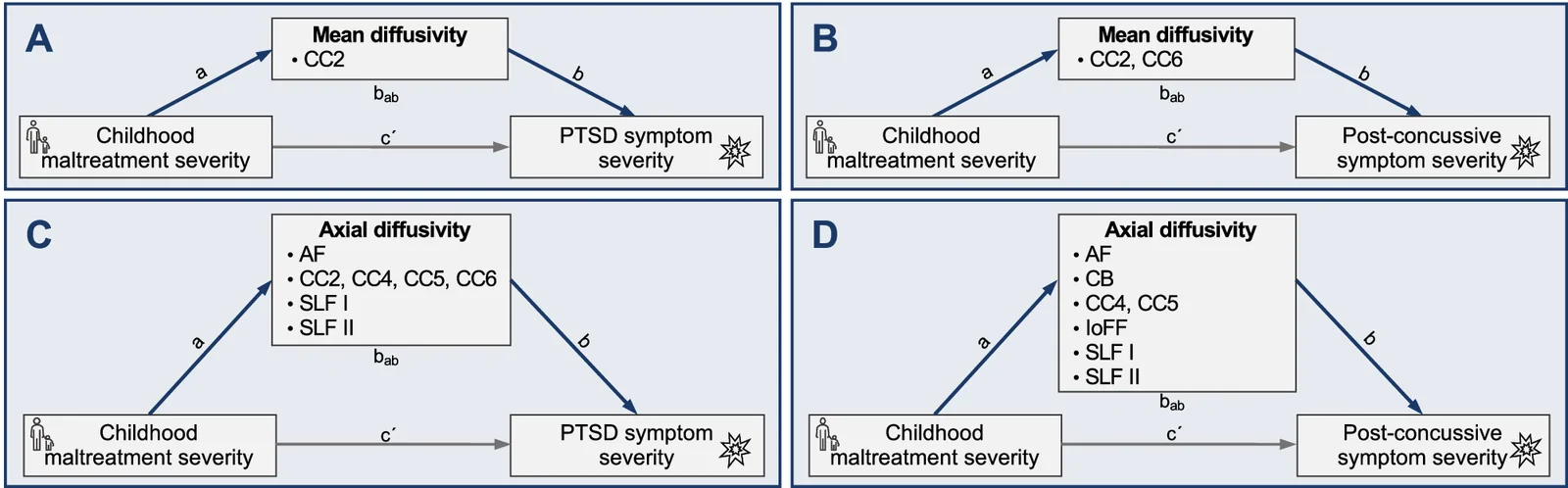
Mediation results between childhood maltreatment severity and post-traumatic symptoms and post-concussive symptoms. This figure illustrates the pattern of similar WM tracts connecting CM severity with post-traumatic symptoms. (**A** and **B**) Mediating effect of MD on the association between CM severity and PTSD or post-concussive symptom severity. (**C** and **D**) Mediating effect of AD on the association between CM severity and PTSD or post-concussive symptom severity. Path a: association between CM severity and WM microstructure; Path b: association between WM microstructure and post-traumatic symptoms; Path c´: association between CM severity and post-traumatic symptoms after accounting for mediating effects of WM microstructure (direct effect); Path ab: association between CM severity and post-traumatic symptoms that is being mediated by WM microstructure (indirect effect). Path ab indicates the extent to which taking WM microstructure into account can explain the effect of CM severity on post-traumatic symptoms. All analyses were adjusted for age and gender. We found a significant association between CM severity and PTSD symptom severity (b_c_ = 0.443, t(299) = 8.583, p < 0.001). When including WM microstructure as a mediator, the relationship between CM severity and PTSD symptom severity was partially mediated by MD in the posterior body region of the CC (CC 6: b_ab_ = 0.022, SE = 0.014, 95% CI = [0.000, 0.054]). We found partially mediating effects of AD in the genu, the anterior and posterior body of the CC (CC 2: b_ab_ = 0.022, SE = 0.012, 95% CI = [0.001, 0.048]; CC4: b_ab_ = 0.025, SE = 0.013, 95% CI = [0.005, 0.055]; CC5: b_ab_ = 0.032, SE = 0.015, 95% CI = [0.010, 0.068]; CC6: b_ab_ = 0.034, SE = 0.014, 95% CI = [0.010, 0.063]), the superior longitudinal fasciculus I (SLF I left: b_ab_ = 0.028, SE = 0.014, 95% CI = [0.005, 0.060]; SLF I right: b_ab_ = 0.024, SE = 0.013, 95% CI = [0.004, 0.053]), and the SLF II (SLF II left: b_ab_ = 0.027, SE = 0.014, 95% CI = [0.003, 0.060]; SLF II right: b_ab_ = 0.024, SE = 0.013, 95% CI = [0.004, 0.053]). We found a significant association between CM severity and post-concussive symptom severity (b_c_ = 0.416, t(148) = 6.048, p < 0.001). When including WM microstructure as a mediator, the relationship between CM severity and PTSD symptom severity was partially mediated by MD in the body region of the CC (CC 2: b_ab_ = 0.028, SE = 0.018, 95% CI = [0.003, 0.073]; CC 6: b_ab_ = 0.040, SE = 0.020, 95% CI = [0.006, 0.085]; CC 7: b_ab_ = 0.025, SE = 0.016, 95% CI = [0.001, 0.061]). We found partially mediating effects of AD in the arcuate fasciculus (AF left: b_ab_ = 0.025, SE = 0.015, 95% CI = [0.002, 0.062]; AF right: b_ab_ = 0.030, SE = 0.016, 95% CI = [0.003, 0.067]), in the cingulum bundle (CB right: b_ab_ = 0.039, SE = 0.019, 95% CI = [0.009, 0.084]), the anterior body region of the CC (CC4: b_ab_ = 0.047, SE = 0.021, 95% CI = [0.015, 0.097]; CC5: b_ab_ = 0.032, SE = 0.018, 95% CI = [0.004, 0.075]), the inferior occipito-frontal fasciculus (IoFF right: b_ab_ = 0.023, SE = 0.015, 95% CI = [0.001, 0.059]), the SLF I (SLF I left: b_ab_ = 0.046, SE = 0.020, 95% CI = [0.014, 0.093]; SLF I right: b_ab_ = 0.032, SE = 0.016, 95% CI = [0.006, 0.069]), and the SLF II (SLF II left: b_ab_ = 0.031, SE = 0.017, 95% CI = [0.004, 0.071]; SLF II right: b_ab_ = 0.030, SE = 0.017, 95% CI = [0.003, 0.067]). *Note*. PTSD post-traumatic stress disorder, CC corpus callosum, AF arcuate fasciculus, SLF superior longitudinal fasciculus, IoFF inferior occipito-frontal fasciculus. *p ≤ 0.05 (two tailed). **p ≤ 0.01 (two tailed). ***p ≤ 0.001.

We found a significant association between CM severity and PTSD symptom severity (Fig. 3 and Table S3, total effect (path c)). When including WM microstructure as a mediator, the relationship between CM severity and PTSD symptom severity was partially mediated by MD of the posterior body region of the CC (Fig. 3 and Table S3, indirect effect (ab)). Moreover, we found partially mediating effects of AD in the genu, the anterior and posterior body of the CC, the SLF I, and the SLF II.

We also identified a significant association between CM severity and post-concussive symptom severity (Fig. 3 and Table S4, total effect (path c)). This relationship was partially mediated by MD of the genu and the posterior body region of the CC (Fig. 3 and Table S4, indirect effect (ab)). Additionally, we observed partially mediating effects of AD of the left and right arcuate fasciculus, the right cingulum bundle, the genu, the anterior and posterior body of the CC, the SLF I, and the SLF II (Fig. 3 and Table S4, indirect effect (ab)). Subsequent adjustments for race and alcohol abuse did not change the significance of most of the significant mediating effects (Table S2).

## Discussion

This study investigated the association between CM severity and adult WM microstructure in the context of the common military conditions PTSD and mTBI. We found higher CM severity to be significantly associated with lower MD and AD of major WM tracts, suggesting a link between CM and adult axonal microstructure. The association between CM and WM microstructure was not moderated by PTSD and/or mTBI diagnoses and thus remained independent of these common comorbidities. Further, WM microstructure mediated the association between CM severity and PTSD and post-concussive symptom severity, particularly in the CC, SLF I, and SLF II, all brain regions involved in sensory integration and language processing. Taken together, results from this study underscore the role of WM microstructure as a potential neurobiological pathway through which CM might predispose to the development of post-traumatic symptoms.

### Associations between childhood maltreatment severity and white matter microstructure

We found significant associations between higher CM severity with lower MD and lower AD of various WM tracts, including the right arcuate fasciculus, the genu and posterior body region of the CC, the ILF, the left inferior occipito-frontal fasciculus, and the right uncinate fasciculus. Our observations in these brain regions are consistent with previous studies that report significant associations between CM and alterations in adult WM microstructure [5].

One of the most consistent findings in individuals with CM is microstructural alterations of the CC [8, 9]. The CC is the largest fiber bundle of the human brain, interconnecting distant regions of both hemispheres [78]. It is essential for functions like sensory integration, attention, language, and memory [14, 15]. Our findings align with previous research, highlighting that the body region of the CC in particular appears to be associated with CM [79]. One possible explanation for this observation could be the rostral-caudal maturation of the CC, with the body region exhibiting prominent growth from childhood to early adulthood, thus potentially rendering it more susceptible to alterations during this developmental period [80]. Another possible explanation could be rooted in the connections of the CC body region with limbic subcortical nuclei [81]. The distinct alterations observed in the body region might be linked to disturbances in the cortico-limbic pathway, stemming from or contributing to emotional distress.

Moreover, we found associations between CM severity and the SLF I and SLF II. These large association fiber bundles connect cortical regions crucial for executive functioning, emotional regulation, and language processing, especially given their links to the Broca and Wernicke regions [82, 83]. SLF I and SLF II are major language processing pathways, showing associations with verbal forms of abuse and neglect [10–12], consistent with our findings (Tables S9-S11).

Previous reports primarily noted associations between CM severity and lower WM FA. However, these previous studies did not correct for partial volume effects. In our study, we applied free-water imaging to account for partial volume with extracellular free-water and identified significant associations between higher CM severity and lower MD and lower AD. In most regions, there were either associations with AD or with both AD and MD, suggesting that mainly AD may be associated with CM. The absence of associations between CM and WM FA in our study could, thus, suggest that FA may not be sensitive to nuanced alterations specific to CM. Conversely, MD is a scalar measure that does not differentiate directions of diffusion. Specifically, MD can serve as a proxy for cellularity and tissue density, and may be influenced by inflammation and edema [69]. In this regard, AD and RD may provide complementary information about microstructural features related to axonal integrity [84, 85] and myelination [70–72], respectively, although these metrics remain indirect measures.

Severity of CM has previously been linked to lower AD of major WM tracts in adulthood in a study of individuals diagnosed with bipolar disorder [10]. Our work aligns well with these findings. AD changes are thought to reflect axonal changes, possibly reflecting altered axonal diameter and increased tortuosity of WM tracts [86, 87]. Of note, in typically developing individuals, AD is observed to increase with WM maturation across childhood and early adolescence [73, 88]. Our observations may thus point to disruptions in axonal development possibly persisting into adulthood. It is nonetheless worth noting that other studies have reported either stable or declining AD throughout development, warranting cautious interpretation [89, 90].

In summary, our observations of both lower MD and lower AD in WM tracts point towards potential axonal alterations associated with CM.

### Moderating effect of PTSD and/or mTBI on the association between childhood maltreatment severity and white matter microstructure

We did not find any moderating effects of current PTSD and/or mTBI diagnoses on the association between CM severity and WM microstructure. This observation may initially appear counterintuitive, given the well-established associations of both PTSD and mTBI with CM [91, 92], as well as with WM microstructure [21]. However, our findings align well with a growing body of research, suggesting that some WM alterations that were previously directly attributed to psychiatric diagnoses in adulthood might rather be associated with a history of CM. For example, Olson et al. [13] observed significant associations between CM and ILF microstructure, independent of a current diagnosis of PTSD, complementing our findings on SLF microstructure. This finding also aligns with studies involving individuals with major depressive disorder, where associations between brain abnormalities such as hippocampal atrophy [93] or WM microstructure alterations [94] and depressive symptoms were no longer significant when controlling for a history of CM. Finally, another study demonstrated that maltreated adolescents with psychiatric comorbidities, including depression, conduct disorder, and attention-deficit/hyperactivity disorder, did not display unique WM microstructural patterns compared to those without psychiatric comorbidities [95].

In summary, our results suggest that the observed relationship between CM and WM microstructure is not significantly influenced by the presence of PTSD or mTBI in adulthood.

### Mediating effect of white matter microstructure on the association between childhood maltreatment severity and post-traumatic symptoms

Given the observed significant association between CM severity and adult WM microstructure and established associations between CM and post-traumatic symptoms, we investigated the mediating effect of WM alterations on the associations between CM severity and post-traumatic symptoms. We observed a mediating association of lower MD and AD of WM tracts, specifically the body of the CC, SLF I, and SLF II. Our findings add to the limited research on the mediating role of WM microstructure between a history of CM and adult mental health symptoms [7], suggesting WM alterations as an underlying pathophysiology.

Interestingly, we identified similar WM tracts connecting CM to both PTSD and post-concussive symptom severity. Post-concussive symptoms often coincide with psychiatric comorbidities, particularly PTSD, making it challenging to fully disentangle post-concussive complaints from symptoms that are also characteristic of PTSD [96]. Moreover, the mental health status before mTBI may predict persistent post-concussive symptoms in adults [97]. In this regard, CM may contribute to this state. In our study, we could not fully disentangle the effects of mTBI from PTSD, as either could be related to CM. However, our findings suggest a possible shared neurobiological foundation that may stem from the lasting impacts of CM.

In summary, WM alterations constitute a key pathophysiological mechanism linking history of CM to post-traumatic symptoms in adulthood.

## Limitations

First, the cross-sectional study design prevents the interpretation of causal relationships. Additionally, we could not assess the role of developmental timing of trauma because the age when participants experienced CM could not be reliably determined. Future longitudinal work is needed to investigate WM variability with more granular information on trauma exposure across development. Future research may also benefit from instruments such as the MACE [98], which provide detailed information on the type, timing, and chronicity of CM.

Second, our research relied on retrospective self-report to identify CM, which may be affected by recall biases [99]. Nonetheless, prevailing evidence suggests that retrospective self-reports of CM are more inclined towards underreporting than over-reporting [100]. In addition, while there is a discrepancy between retrospective and prospective self-reports of CM [99], retrospective self-report of CM appears to be a better indicator of adult psychopathology, irrespective of type of CM assessment [101, 102]. Such evidence underscores the reliability and clinical meaningfulness of the current analyses.

Third, the specific characteristics of PTSD and mTBI could not thoroughly be accounted for in our study. Participants showed symptoms that may have been related to a variety of traumatic events. In addition, the perception of the severity of trauma may also play a role in the heterogeneity of traumatic events. Future studies need to consider the heterogeneity when evaluating trauma.

Fourth, the PTSD-only subgroup was small and disproportionate to other groups, reducing power for PTSD-specific contrasts and moderation tests. While the primary associations estimated in the full sample remain interpretable, PTSD-specific moderation results should be interpreted with caution.

Fifth, PTSD diagnoses were based on DSM IV criteria. Although DSM IV and DSM 5 overlap substantially, generalizability to DSM 5 definitions may be limited; replication with DSM 5-based datasets is warranted.

Sixth, CTQ validity scoring could not be performed because item-level data were unavailable. While this is a limitation, prior studies indicate CTQ subscales retain reliability and clinical utility in predicting adult psychopathology even without validity scoring [30, 31]. Moreover, our use of plausibility and robustness checks mitigates concerns about bias from skew or floor effects.

## Conclusion

Findings from this research study suggest that a history of CM is associated with WM microstructure independently of PTSD and/or mTBI diagnosis. Microstructural alterations of major WM tracts mediate the relationship between CM and symptom severity in adulthood. Specifically, the CC, the SLF I, and SLF II, previously associated with sensory integration and language processing, show significant mediation effects. Results from this study thus further suggest that microstructural alterations of white matter tracts may constitute a key pathomechanism underlying the association between a history of CM and adult psychopathology.

## Supplementary information

Supplemental Material

## Data Availability

Due to data use restrictions, the original dataset cannot be shared. To facilitate reproducibility, a synthetic dataset is provided alongside the analysis code.

## Code availability

Analysis code and synthetic data are publicly available at https://doi.org/10.5281/zenodo.18985846. The repository includes an R project with all analysis scripts and a synthetic dataset to allow computational reproducibility of the workflow.
