# Supplementary material for: The role of white matter microstructure in the association between childhood maltreatment and adult post-traumatic symptoms
Source: Mol Psychiatry. 2026 Oct 10;31(12):6701–10. doi: 10.1038/s41380-026-03624-1 (PMC13736700; doi:10.1038/s41380-026-03624-1)
Supplement: Supplementary file 1 [file 41380_2026_3624_MOESM1_ESM.pdf]

The Role of White Matter Microstructure in the Association between Childhood Maltreatment and Adult Post-Traumatic Symptoms

Online Supplementary Material

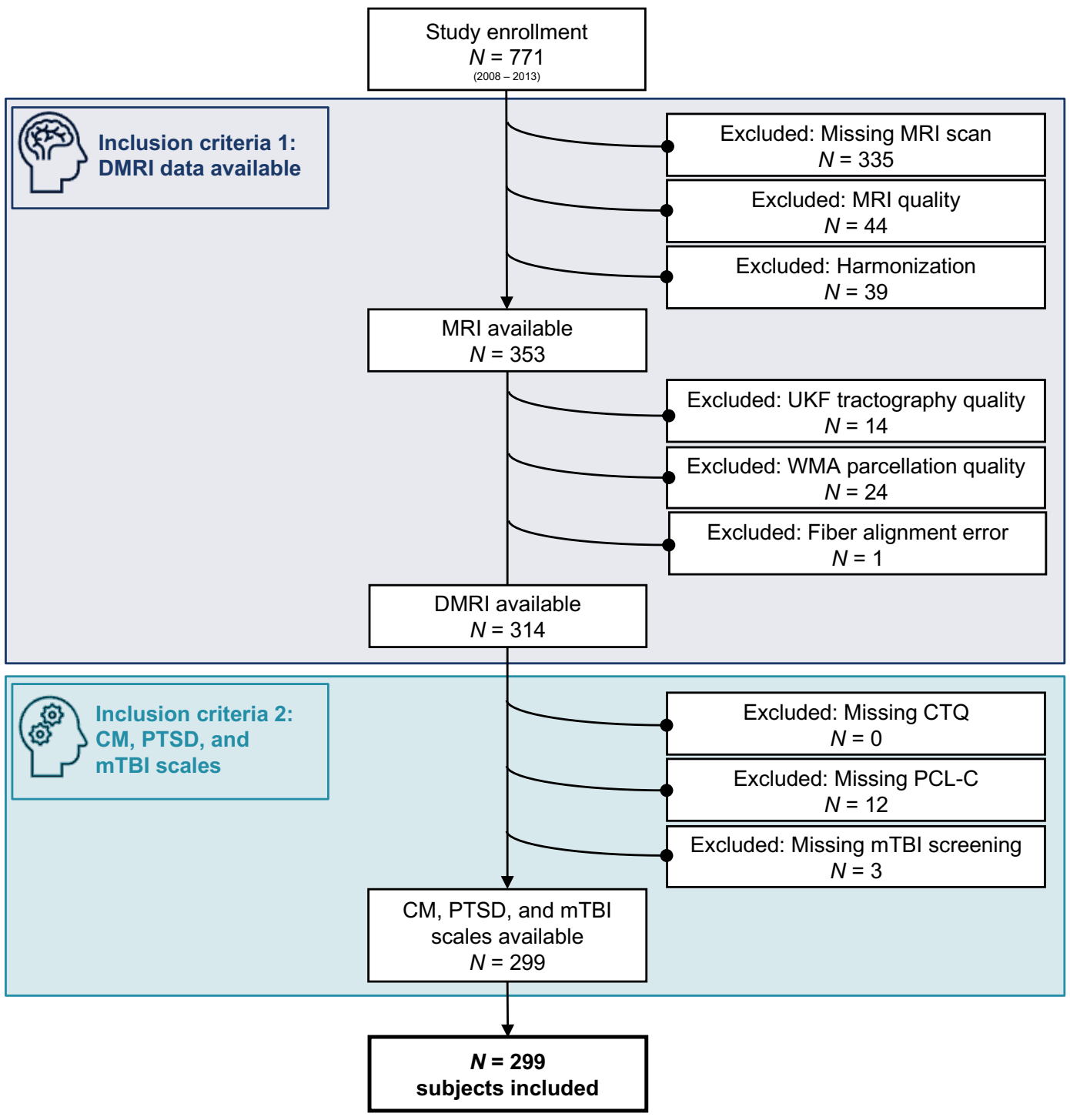

Figure S1. Flowchart of the recruitment procedure.
Note. DMRI = diffusion magnetic resonance imaging; MRI = magnetic resonance imaging; UKF; UKF = unscented Kalman filter; WMA = white matter analysis; CTQ = Childhood Trauma Questionnaire; PCL-C = PTSD Checklist–Civilian Version; INTRuST = Injury and Traumatic Stress Screening Tool; CM = childhood maltreatment; PTSD = post-traumatic stress disorder; mTBI = mild traumatic brain injury. This figure illustrates the hierarchical recruitment approach.

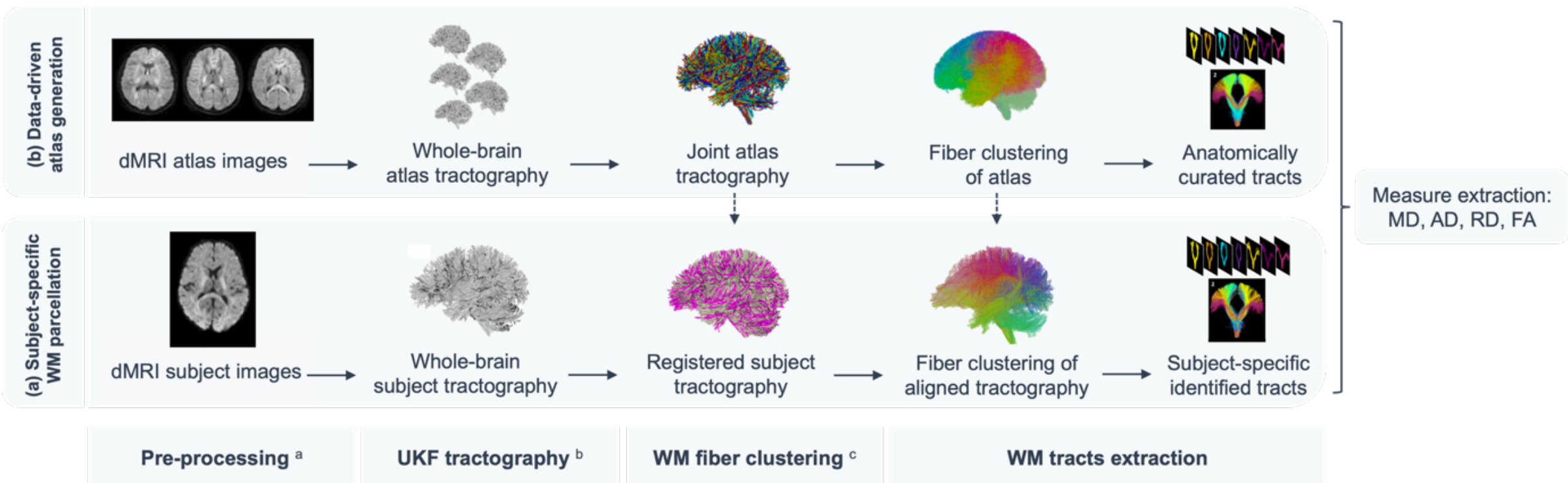

Figure S2. Neuroimaging processing flow.
Note. dMRI, diffusion-weighted magnetic resonance imaging; WM; white matter; UKF, Unscented Kalman Filter; MD, mean diffusivity; AD, axial diffusivity; RD, radial diffusivity; FA, fractional anisotropy. Subfigure (a) visualizes the process of the data-driven fiber-clustering atlas generation that is then utilized for the whole-brain white matter parcellation for a new subject, as depicted in subfigure (b).
[a] https://github.com/pnlbwh/pnlutil/blob/master/pipeline
[b] https://github.com/pnlbwh/ukftractography
[c] https://github.com/SlicerDMRI/whitematteranalysis
Adapted from "An anatomically curated fiber clustering white matter atlas for consistent white matter tract parcellation across the lifespan" by Zhang et al., 2018, *Neuroimage*. 179, p. 432 (doi:10.1016/j.neuroimage.2018.06.027).

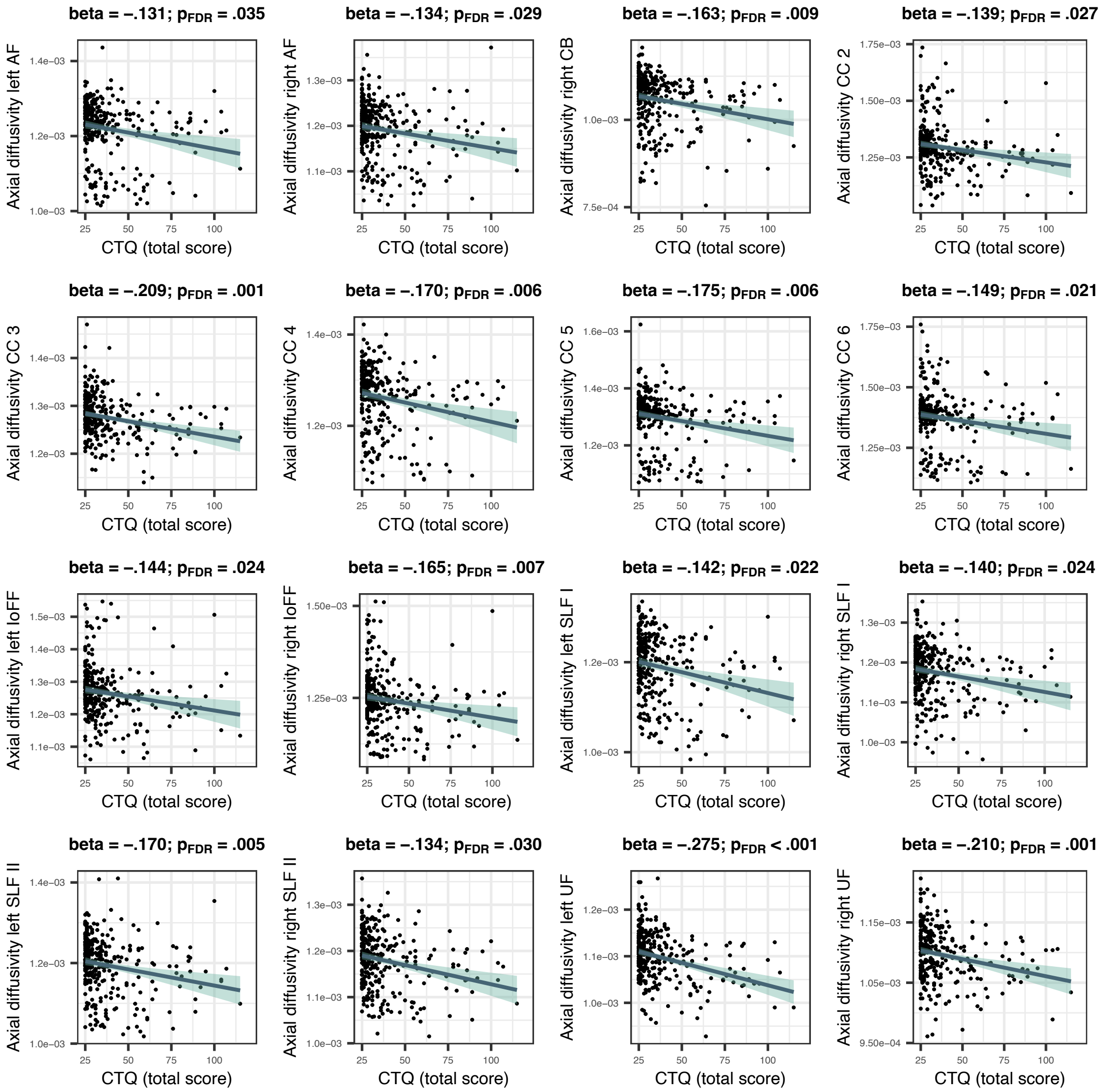

Figure S3. Significant associations between childhood maltreatment severity and axial diffusivity. *Note.* FDR, false-discovery rate; CTQ, Childhood Trauma Questionnaire; AF, arcuate fasciculus; CB, cingulum bundle; CC, corpus callosum; IoFF, inferior occipito-frontal fasciculus; SLF, superior longitudinal fasciculus; UF, uncinate fasciculus.

Table S1. Diffusion-weighted magnetic resonance imaging acquisition parameters.

Parameter	GE	SIEMENS	PHILIPS
Orientation	axial	axial	axial
Phase encoding direction	left/right	anterior/posterior	anterior/posterior
Field of view (in mm)	256	256	256
Bandwidth (in kHz or Hz/Px)	250	1396	1271
Number of directions	86	87	64
b-value	900	900	900
Number of b0	1	0	7
Matrix size	128x128	128x128	128x128
Voxel size (in mm^3)	2x2x2	2x2x2	2x2x2
Number of slices	73	73	73
Acquisition time (in min)	14:40	14:08	14:21

Note. Multi-site study; MRI data acquisition on Tim Trio, Siemens Healthineers, Erlangen, Germany; GE 750, GE Healthcare, Chicago, IL, USA; Achieva, Philips Healthcare, Best, Netherlands.

Table S2. Comparison of multiple regression and mediation results with race and alcohol abuse as further covariates.

Covariates		**Age, gender**			**Age, gender, race**			**Age, gender, race, alcohol abuse**		
		Sig. Path a (*N* = 299)	Sig. Mediation PTSD (*N* = 299)	Sig. Mediation PCS (*N* = 177)	Sig. Path a (*N* = 288)	Sig. Mediation PTSD (*N* = 288)	Sig. Mediation PCS (*N* = 173)	Sig. Path a (*N* = 283)	Sig. Mediation PTSD (*N* = 283)	Sig. Mediation PCS (*N* = 171)
**MD**	**AF right**	✓	✗	✗	✓	✗	✗	✗	✗	✗
	**CC 1**	✓	✗	✗	✓	✗	✗	✓	✗	✗
	**CC 2**	✓	✗	✓	✓	✓	✓	✓	✓	✓
	**CC 6**	✓	✓	✓	✓	✓	✓	✓	✓	✓
	**ILF right**	✓	✗	✗	✓	✗	✗	✓	✗	✗
	**IoFF left**	✓	✗	✗	✓	✗	✗	✓	✗	✗
	**IoFF right**	✓	✗	✗	✓	✗	✓	✓	✓	✓
	**UF right**	✓	✗	✗	✓	✗	✓	✓	✗	✓
**AD**	**AF left**	✓	✓	✓	✓	✓	✓	✓	✓	✓
	**AF right**	✓	✗	✓	✗	✗	✓	✓	✗	✓
	**CB right**	✓	✗	✓	✓	✗	✓	✓	✗	✓
	**CC 2**	✓	✓	✗	✓	✓	✗	✓	✓	✗
	**CC 3**	✓	✓	✗	✓	✓	✗	✓	✓	✗
	**CC 4**	✓	✓	✓	✓	✓	✓	✓	✓	✓
	**CC 5**	✓	✓	✓	✓	✓	✓	✓	✓	✓
	**CC 6**	✓	✓	✗	✓	✓	✗	✓	✓	✗
	**IoFF left**	✓	✗	✗	✓	✓	✗	✓	✓	✗
	**IoFF right**	✓	✗	✓	✓	✗	✓	✓	✗	✓
	**SLF I left**	✓	✓	✓	✓	✓	✓	✓	✓	✓
	**SLF I right**	✓	✓	✓	✓	✓	✓	✓	✓	✓
	**SLF II left**	✓	✓	✓	✓	✓	✓	✓	✓	✓
	**SLF II right**	✓	✓	✓	✓	✓	✓	✓	✓	✓
	**SLF III right**	✓	✓	✗	✓	✗	✗	✓	✓	✗
	**UF left**	✓	✗	✗	✓	✗	✗	✓	✗	✗
	**UF right**	✓	✗	✗	✓	✗	✗	✓	✗	✗

Note. MD, mean diffusivity; AD, axial diffusivity; AF, arcuate fasciculus; CB, cingulum bundle; CC, corpus callosum; ILF, inferior longitudinal fasciculus; IoFF, inferior occipito-frontal fasciculus; SLF, superior longitudinal fasciculus; UF, uncinate fasciculus; Sig., significant; PTSD, post-traumatic stress disorder; PCS, post-concussive symptoms. Sig. path a: association between childhood maltreatment severity and white matter tracts; Sig. mediation PTSD: association between childhood maltreatment severity and PTSD symptom severity that is being mediated by white matter microstructure. Sig. mediation PCS: association between childhood maltreatment severity and post-concussive symptom severity that is being mediated by white matter microstructure. A check mark on the light green background indicates that path a was significant, while a check mark on the dark green background indicates that the mediation was significant. An X indicates that the path a or the mediation were not significant.

Table S3. Mediation results of childhood maltreatment severity on PTSD symptom severity (*N* = 299).

		**Total effect** *(c)*			**Direct effect** *(c')*			**Indirect effect** *(ab)*			
		b_c	t-value	p-value	$b_{c'}$	t-value	p-value	b_{ab}	SE	Percentile bootstrap 95% confidence interval	
										Lower	Upper
**MD**	**CC 6**	.443	8.583	**< .000**	.421	8.058	**< .000**	.022	.014	**.000**	**.054**
**AD**	**AF left**	.443	8.583	**< .000**	.412	8.206	**< .000**	.020	.012	**.002**	**.049**
	**CC 2**	.443	8.583	**< .000**	.410	8.186	**< .000**	.022	.012	**.001**	**.048**
	**CC 3**	.443	8.583	**< .000**	.408	8.071	**< .000**	.025	.013	**.005**	**.055**
	**CC 4**	.443	8.583	**< .000**	.413	8.048	**< .000**	.019	.011	**.000**	**.045**
	**CC 5**	.443	8.583	**< .000**	.400	7.976	**< .000**	.034	.015	**.010**	**.068**
	**CC 6**	.443	8.583	**< .000**	.401	8.104	**< .000**	.032	.014	**.007**	**.063**
	**SLF I left**	.443	8.583	**< .000**	.404	8.042	**< .000**	.028	.014	**.005**	**.060**
	**SLF I right**	.443	8.583	**< .000**	.409	8.158	**< .000**	.024	.013	**.004**	**.053**
	**SLF II left**	.443	8.583	**< .000**	.405	8.140	**< .000**	.027	.014	**.003**	**.060**
	**SLF II right**	.443	8.583	**< .000**	.408	8.066	**< .000**	.024	.013	**.004**	**.053**

Note. MD, mean diffusivity; AD, axial diffusivity; CC, corpus callosum; SLF, superior longitudinal fasciculus. The association between childhood maltreatment severity and PTSD symptom severity was partially mediated by mean diffusivity and axial diffusivity in various white matter tracts. All analyses were adjusted for age and gender as covariates.

Table S4. Mediation results of childhood maltreatment severity on post-concussive symptom severity (N = 177).

		**Total effect** *(c)*			**Direct effect** *(c')*			**Indirect effect** *(ab)*			
		b_c	t-value	p-value	$b_{c'}$	t-value	p-value	b_{ab}	SE	Percentile bootstrap 95% confidence interval	
										Lower	Upper
**MD**	**CC 2**	.416	6.048	**< .000**	.308	5.500	**< .000**	.028	.018	.003	.073
	**CC 6**	.416	6.048	**< .000**	.297	5.494	**< .000**	.040	.020	.006	.085
**AD**	**AF left**	.416	6.048	**< .000**	.311	5.655	**< .000**	.025	.015	.002	.062
	**AF right**	.416	6.048	**< .000**	.307	5.606	**< .000**	.030	.016	.003	.067
	**CB right**	.416	6.048	**< .000**	.297	5.493	**< .000**	.039	.019	.009	.084
	**CC 4**	.416	6.048	**< .000**	.289	5.327	**< .000**	.047	.021	.015	.097
	**CC 5**	.416	6.048	**< .000**	.304	5.587	**< .000**	.032	.018	.004	.075
	**IoFF right**	.416	6.048	**< .000**	.313	5.634	**< .000**	.023	.015	.001	.059
	**SLF I left**	.416	6.048	**< .000**	.290	5.344	**< .000**	.046	.020	.014	.093
	**SLF I right**	.416	6.048	**< .000**	.377	5.635	**< .000**	.032	.016	.006	.069
	**SLF II left**	.416	6.048	**< .000**	.378	5.564	**< .000**	.031	.017	.004	.071
	**SLF II right**	.416	6.048	**< .000**	.379	5.462	**< .000**	.030	.017	.003	.067

Note. MD, mean diffusivity; AD, axial diffusivity; CC, corpus callosum; AF, arcuate fasciculus, CB, cingulum bundle; SLF, superior longitudinal fasciculus. The association between childhood maltreatment severity and post-concussive symptom severity was partially mediated by mean diffusivity and axial diffusivity in various white matter tracts. All analyses were adjusted for age and gender as covariates.

Supplementary Analyses

Robustness of Regression Analyses

To evaluate robustness, regressions were re-estimated using heteroskedasticity-consistent (HC3) standard errors and bias-corrected accelerated (BCa) bootstrap 95% confidence intervals (5,000 resamples; **Table S5**). To ensure the small PTSD-only subgroup (*n* = 15) did disproportionately influence results, regressions were repeated after excluding these participants (*N* = 287; **Table S6**). Findings were substantively unchanged.

Robustness of Moderation Analyses

Moderation models were re-estimated using HC3 robust standard errors and BCa bootstrap 95% confidence intervals (5,000 resamples). Effects were considered robust if both criteria were met. Interaction effects were generally small and confidence intervals spanned zero, except an isolated PTSD effect in axial diffusivity of CC 6 (**Table S7**). This effect did not replicate across adjacent callosal regions. To address comorbidity more directly, we also estimated a three-way CM×PTSD×mTBI interaction (PROCESS Model 3), which was nonsignificant across tracts (**Table S8**).

Table S5. Multiple regression results between childhood maltreatment severity and white matter microstructure with HC3 standard errors andHC3 and BCa confidence intervals.

	Fractional anisotropy (FA)				Mean diffusivity (MD)				Axial diffusivity (AD)				Radial diffusivity (RD)			
	**beta**	**p_{FDR} (HC3)**	**95% CI(HC3)**	**95% CI (BCa)**	**beta**	**p_{FDR} (HC3)**	**95% CI (HC3)**	**95% CI (BCa)**	**beta**	**p_{FDR} (HC3)**	**95% CI (HC3)**	**95% CI (BCa)**	**beta**	**p_{FDR} (HC3)**	**95% CI (HC3)**	**95% CI (BCa)**
**AF left**	-.052	.545	[-.168, .064]	[-.172, .061]	-.122	.069	[-.235, -.010]	[-.244, -.016]	**-.134**	**.034**	**[-.247, -.022]**	**[-.260, -.033]**	.006	.944	[-.111, .123]	[-.104, .126]
**AF right**	-.005	.958	[-.128, .118]	[-.118, .116]	**-.152**	**.018**	**[-.260, -.045]**	**[-.265, -.051]**	-.131	.091	[-.268, .005]	[-.251, .022]	-.051	.600	[-.169, .067]	[-.179, .058]
**CB left**	-.020	.821	[-.144, .104]	[-.120, .134]	-.050	.508	[-.170, .070]	[-.179, .057]	-.102	.098	[-.210, .006]	[-.213, -.004]	-.020	.837	[-.144, .103]	[-.158, .084]
**CB right**	-.059	.491	[-.171, .053]	[-.164, .059]	-.093	.168	[-.205, .019]	[-.220, .005]	**-.163**	**.012**	**[-.279, -.047]**	**[-.285, -.052]**	-.043	.612	[-.153, .066]	[-.163, .050]
**CC 1**	.006	.949	[-.104, .116]	[-.104, .116]	**-.167**	**.033**	**[-.296, -.038]**	**[-.296, -.039]**	-.107	.170	[-.241, .028]	[-.229, .026]	-.057	.479	[-.162, .047]	[-.168, .041]
**CC 2**	-.003	.973	[-.112, .107]	[-.100, .112]	**-.180**	**.018**	**[-.308, -.053]**	**[-.300, -.049]**	-.139	.056	[-.268, -.010]	[-.249, .000]	-.060	.475	[-.167, .047]	[-.168, .037]
**CC 3**	-.021	.817	[-.144, .102]	[-.134, .108]	-.126	.088	[-.250, -.002]	[-.238, .007]	**-.209**	**.000**	**[-.317, -.100]**	**[-.315, -.095]**	-.042	.672	[-.167, .083]	[-.163, .079]
**CC 4**	-.007	.949	[-.125, .112]	[-.107, .127]	-.118	.066	[-.226, -.011]	[-.237, -.019]	**-.170**	**.006**	**[-.282, -.058]**	**[-.296, -.069]**	-.052	.578	[-.164, .061]	[-.186, .044]
**CC 5**	-.036	.675	[-.150, .078]	[-.139, .095]	**-.132**	**.040**	**[-.238, -.027]**	**[-.251, -.037]**	**-.175**	**.007**	**[-.291, -.059]**	**[-.295, -.074]**	-.029	.732	[-.138, .079]	[-.158, .061]
**CC 6**	-.046	.623	[-.173, .081]	[-.158, .095]	**-.193**	**.006**	**[-.313, -.073]**	**[-.306, -.074]**	**-.149**	**.030**	**[-.272, -.027]**	**[-.275, -.030]**	-.023	.821	[-.144, .099]	[-.169, .078]
**CC 7**	-.052	.576	[-.178, .074]	[-.156, .096]	-.129	.077	[-.252, -.007]	[-.251, -.010]	-.085	.260	[-.214, .043]	[-.199, .068]	-.003	.976	[-.122, .117]	[-.149, .097]
**ILF left**	.033	.732	[-.088, .154]	[-.069, .171]	-.078	.344	[-.213, .058]	[-.203, .062]	-.050	.556	[-.187, .088]	[-.166, .113]	-.053	.601	[-.179, .074]	[-.191, .057]
**ILF right**	.024	.792	[-.095, .144]	[-.079, .159]	**-.144**	**.044**	**[-.263, -.025]**	**[-.269, -.030]**	-.115	.111	[-.242, .012]	[-.238, .007]	-.080	.342	[-.197, .037]	[-.214, .021]
**IoFF left**	-.022	.798	[-.135, .091]	[-.120, .114]	**-.196**	**.011**	**[-.325, -.066]**	**[-.315, -.061]**	-.144	.059	[-.278, -.009]	[-.257, -.004]	-.047	.601	[-.158, .064]	[-.169, .054]
**IoFF right**	-.030	.751	[-.152, .093]	[-.151, .086]	**-.161**	**.020**	**[-.276, -.045]**	**[-.280, -.055]**	-.123	.091	[-.251, .005]	[-.234, .031]	-.026	.788	[-.144, .093]	[-.145, .085]
**SLF I left**	-.044	.623	[-.163, .076]	[-.158, .081]	-.120	.051	[-.222, -.018]	[-.228, -.025]	**-.165**	**.012**	**[-.282, -.048]**	**[-.278, -.048]**	-.049	.578	[-.155, .058]	[-.162, .049]
**SLF I right**	-.054	.545	[-.171, .063]	[-.179, .053]	-.085	.165	[-.185, .015]	[-.193, .005]	**-.142**	**.016**	**[-.248, -.036]**	**[-.252, -.040]**	-.017	.837	[-.124, .089]	[-.126, .083]
**SLF II left**	-.075	.379	[-.192, .042]	[-.189, .037]	-.072	.233	[-.174, .029]	[-.178, .021]	**-.140**	**.036**	**[-.260, -.021]**	**[-.245, -.005]**	.028	.752	[-.084, .140]	[-.084, .133]
**SLF II right**	-.076	.365	[-.191, .040]	[-.192, .034]	-.100	.116	[-.206, .006]	[-.205, .007]	**-.170**	**.002**	**[-.272, -.069]**	**[-.269, -.068]**	.011	.899	[-.103, .124]	[-.105, .119]
**SLF III left**	-.022	.803	[-.142, .097]	[-.142, .102]	-.095	.189	[-.218, .028]	[-.221, .020]	-.077	.352	[-.217, .062]	[-.176, .102]	-.012	.899	[-.130, .106]	[-.128, .101]
**SLF III right**	-.061	.504	[-.181, .059]	[-.175, .062]	-.104	.168	[-.228, .021]	[-.237, .010]	-.134	.066	[-.264, -.005]	[-.254, .009]	.019	.837	[-.101, .138]	[-.107, .132]
**UF left**	-.053	.545	[-.169, .063]	[-.164, .072]	-.124	.062	[-.235, -.014]	[-.235, -.019]	**-.275**	**.000**	**[-.386, -.163]**	**[-.380, -.166]**	-.030	.732	[-.140, .081]	[-.146, .071]
**UF right**	.025	.792	[-.097, .147]	[-.075, .166]	**-.144**	**.037**	**[-.257, -.031]**	**[-.269, -.042]**	**-.210**	**.000**	**[-.318, -.102]**	**[-.322, -.112]**	-.077	.349	[-.192, .037]	[-.205, .024]

Note. FA, fractional anisotropy; MD, mean diffusivity; AD, axial diffusivity; RD, radial diffusivity; FDR, false-discovery rate; AF, arcuate fasciculus; CB, cingulum bundle; CC, corpus callosum; ILF, inferior longitudinal fasciculus; IoFF, inferior occipito-frontal fasciculus; SLF, superior longitudinal fasciculus; UF, uncinate fasciculus. Standardized regression coefficients (beta) represent associations between childhood maltreatment severity and tract diffusivity. All analyses were corrected for age and gender as covariates. pFDR (HC3) = Benjamini–Hochberg false discovery rate–adjusted p-values using heteroskedasticity-consistent (HC3) standard errors. 95% CIs are reported for both HC3-based t intervals and bias-corrected and accelerated (BCa) bootstrap intervals (5,000 resamples).

Table S6. Multiple regression results between childhood maltreatment severity and white matter microstructure without PTSD subgroup (*N* = 287).

	Fractional anisotropy (FA)		Mean diffusivity (MD)		Axial diffusivity (AD)		Radial diffusivity (RD)	
	p_{FDR}	beta	p_{FDR}	beta	p_{FDR}	beta	p_{FDR}	beta
**AF left**	.195	-.097	.271	-.082	**.016**	**-.152**	.473	.060
**AF right**	.483	-.049	.143	-.112	**.017**	**-.151**	.990	.002
**CB left**	.401	-.058	.983	-.001	.411	-.058	.827	.024
**CB right**	.149	-.108	.686	-.031	.077	-.117	.864	.020
**CC 1**	.484	-.048	**.049**	**-.144**	**.021**	**-.150**	.990	-.001
**CC 2**	.364	-.063	**.021**	**-.166**	**.005**	**-.182**	.988	-.005
**CC 3**	.347	-.068	.233	-.093	**.001**	**-.213**	.990	.002
**CC 4**	.347	-.069	.267	-.084	**.007**	**-.173**	.990	.001
**CC 5**	.260	-.084	.176	-.106	**.004**	**-.187**	.904	.016
**CC 6**	.128	-.115	**.010**	**-.180**	**.003**	**-.194**	.592	.048
**CC 7**	.070	-.133	**.020**	**-.169**	**.015**	**-.161**	.389	.075
**ILF left**	.629	-.032	.578	-.044	.167	-.093	.958	.009
**ILF right**	.496	-.047	.148	-.112	**.017**	**-.155**	.911	-.015
**IoFF left**	.182	-.099	**.021**	**-.167**	**.002**	**-.200**	.729	.034
**IoFF right**	.253	-.085	**.040**	**-.151**	**.006**	**-.177**	.729	.033
**SLF I left**	.347	-.069	.109	-.119	**.004**	**-.182**	.729	-.035
**SLF I right**	.142	-.111	.551	-.047	**.023**	**-.142**	.729	.033
**SLF II left**	.217	-.093	.444	-.060	**.016**	**-.150**	.585	.050
**SLF II right**	.142	-.108	.440	-.061	**.007**	**-.168**	.565	.052
**SLF III left**	.253	-.084	.242	-.089	**.017**	**-.150**	.585	.050
**SLF III right**	.117	-.115	.242	-.089	**.003**	**-.189**	.389	.074
**UF left**	.342	-.070	.242	-.091	**.000**	**-.248**	.990	-.004
**UF right**	.757	-.021	.264	-.085	**.008**	**-.174**	.823	-.025

Higher childhood maltreatment severity was significantly associated with lower mean diffusivity and axial diffusivity in various white matter tracts. No significant associations were found with fractional anisotropy or radial diffusivity. All analyses were corrected for age and gender as covariates.
Note. FA, fractional anisotropy; MD, mean diffusivity; AD, axial diffusivity; RD, radial diffusivity; FDR, false-discovery rate; AF, arcuate fasciculus; CB, cingulum bundle; CC, corpus callosum; ILF, inferior longitudinal fasciculus; IoFF, inferior occipito-frontal fasciculus; SLF, superior longitudinal fasciculus; UF, uncinate fasciculus.

Table S7. Moderation Effects with HC3 Standard Errors and Bootstrap Confidence Intervals

	Tract	Moderator	beta	p (HC3)	95% CI (HC3)	95% CI (BCa)
**MD**	AF right	mTBI	-0.011	.836	[-.230, .186]	[-.247, .182]
	AF right	PTSD	-0.028	.527	[-.523, .268]	[-.728, .271]
	AF right	PTSD+mTBI	0.012	.837	[-.287, .354]	[-.279, .339]
	CC 1	mTBI	-0.012	.876	[-.338, .288]	[-.288, .291]
	CC 1	PTSD	-0.001	.989	[-.478, .471]	[-.572, .491]
	CC 1	PTSD+mTBI	-0.039	.537	[-.473, .247]	[-.454, .247]
	CC 2	mTBI	-0.014	.873	[-.379, .322]	[-.284, .311]
	CC 2	PTSD	0.019	.649	[-.291, .467]	[-.347, .484]
	CC 2	PTSD+mTBI	0.022	.647	[-.213, .343]	[-.184, .349]
	CC 6	mTBI	-0.014	.853	[-.343, .284]	[-.300, .280]
	CC 6	PTSD	-0.012	.755	[-.401, .291]	[-.440, .275]
	CC 6	PTSD+mTBI	0.076	.155	[-.084, .527]	[-.097, .493]
	ILF right	mTBI	0.013	.831	[-.230, .285]	[-.205, .297]
	ILF right	PTSD	-0.025	.556	[-.501, .270]	[-.688, .312]
	ILF right	PTSD+mTBI	0.070	.221	[-.122, .527]	[-.108, .519]
	IoFF left	mTBI	0.020	.796	[-.276, .360]	[-.217, .373]
	IoFF left	PTSD	-0.030	.577	[-.621, .347]	[-.596, .344]
	IoFF left	PTSD+mTBI	0.015	.752	[-.228, .316]	[-.217, .326]
	IoFF right	mTBI	0.010	.872	[-.223, .263]	[-.236, .248]
	IoFF right	PTSD	0.021	.668	[-.352, .548]	[-.423, .569]
	IoFF right	PTSD+mTBI	0.029	.528	[-.175, .341]	[-.169, .316]
	UF right	mTBI	0.008	.879	[-.208, .242]	[-.193, .252]
	UF right	PTSD	-0.047	.358	[-.676, .245]	[-.760, .236]
	UF right	PTSD+mTBI	0.022	.571	[-.158, .286]	[-.138, .311]
**AD**	AF left	mTBI	-0.014	.820	[-.283, .225]	[-.292, .210]
	AF left	PTSD	0.039	.337	[-.186, .540]	[-.183, .595]
	AF left	PTSD+mTBI	0.030	.569	[-.216, .392]	[-.246, .373]
	AF right	mTBI	-0.015	.828	[-.313, .251]	[-.333, .223]
	AF right	PTSD	0.021	.749	[-.484, .672]	[-.310, .888]
	AF right	PTSD+mTBI	0.044	.428	[-.189, .445]	[-.199, .431]
	CB right	mTBI	-0.047	.430	[-.344, .147]	[-.319, .135]
	CB right	PTSD	-0.047	.354	[-.678, .243]	[-.806, .184]
	CB right	PTSD+mTBI	0.083	.052	[-.003, .482]	[.013, .493]
	CC 2	mTBI	-0.015	.822	[-.308, .244]	[-.273, .232]
	CC 2	PTSD	0.081	.145	[-.129, .867]	[.077, 1.060]
	CC 2	PTSD+mTBI	-0.001	.978	[-.252, .245]	[-.285, .218]
	CC 3	mTBI	-0.065	.300	[-.391, .121]	[-.354, .135]
	CC 3	PTSD	0.053	.077	[-.026, .509]	[-.041, .524]
	CC 3	PTSD+mTBI	0.032	.527	[-.196, .382]	[-.214, .341]
	CC 4	mTBI	-0.054	.416	[-.387, .161]	[-.414, .128]
	CC 4	PTSD	0.026	.398	[-.158, .397]	[-.194, .444]
	CC 4	PTSD+mTBI	0.052	.378	[-.185, .486]	[-.207, .452]
	CC 5	mTBI	0.002	.975	[-.284, .293]	[-.252, .294]
	CC 5	PTSD	0.043	.107	[-.043, .440]	[-.045, .516]
	CC 5	PTSD+mTBI	0.038	.509	[-.217, .437]	[-.227, .429]
	CC 6	mTBI	-0.010	.890	[-.313, .272]	[-.277, .265]
	CC 6	PTSD	**0.062**	**.043**	**[.009, .561]**	**[.031, .622]**
	CC 6	PTSD+mTBI	0.039	.432	[-.172, .401]	[-.208, .378]
	IoFF left	mTBI	-0.020	.772	[-.325, .241]	[-.291, .219]
	IoFF left	PTSD	0.076	.228	[-.219, .913]	[-.064, .983]
	IoFF left	PTSD+mTBI	-0.012	.797	[-.312, .240]	[-.344, .218]
	SLF I left	mTBI	-0.051	.407	[-.360, .146]	[-.345, .147]
	SLF I left	PTSD	0.060	.190	[-.137, .688]	[-.162, .780]
	SLF I left	PTSD+mTBI	0.055	.296	[-.141, .463]	[-.155, .438]
	SLF I right	mTBI	-0.049	.353	[-.320, .114]	[-.333, .104]

Table S7. Moderation Effects with HC3 Standard Errors and Bootstrap Confidence Intervals

Tract	Moderator	beta	p (HC3)	95% CI (HC3)	95% CI (BCa)
SLF I right	PTSD	0.022	.608	[-.288, .491]	[-.443, .461]
SLF I right	PTSD+mTBI	0.055	.186	[-.078, .398]	[-.072, .394]
SLF II left	mTBI	-0.009	.884	[-.273, .235]	[-.286, .220]
SLF II left	PTSD	0.044	.434	[-.302, .701]	[-.151, .893]
SLF II left	PTSD+mTBI	0.001	.982	[-.295, .302]	[-.313, .293]
SLF II right	mTBI	0.003	.958	[-.217, .229]	[-.196, .228]
SLF II right	PTSD	0.024	.554	[-.253, .471]	[-.249, .492]
SLF II right	PTSD+mTBI	0.007	.865	[-.230, .274]	[-.248, .253]
SLF III right	mTBI	-0.018	.773	[-.296, .221]	[-.314, .199]
SLF III right	PTSD	0.066	.239	[-.203, .809]	[-.008, .953]
SLF III right	PTSD+mTBI	-0.019	.718	[-.361, .249]	[-.357, .236]

Note. MD, mean diffusivity; AD, axial diffusivity; AF, arcuate fasciculus; CB, cingulum bundle; CC, corpus callosum; ILF, inferior longitudinal fasciculus; IoFF, inferior occipito-frontal fasciculus; SLF, superior longitudinal fasciculus; UF, uncinate fasciculus; PTSD, post-traumatic stress disorder; mTBI, mild traumatic brain injury; HC3, heteroskedasticity-consistent; CI, confidence interval; BCa, bias-corrected accelerated. Moderation models tested whether the association between childhood maltreatment severity and white matter microstructure was moderated by PTSD, mTBI, and PTSD+mTBI, adjusting for age and sex. Reported are standardized interaction coefficients (beta), HC3 p-values, and BCa bootstrap 95% confidence intervals (5,000 resamples). Estimates provide robustness checks for potential heteroskedasticity and unequal group sizes. All analyses were corrected for age and gender.

Table S8. Sensitivity analysis of three-way CM × PTSD × mTBI interactions on white matter microstructure.

	Tract	R^2	beta	p (HC3)	95% CI (HC3)	95% CI (BCa)
**MD**	AF right	.074	.134	= .627	[-.406, .674]	[-.434, .760]
	CC 1	.063	-.059	= .856	[-.702, .583]	[-.708, .680]
	CC 2	.071	.030	= .917	[-.527, .586]	[-.516, .613]
	CC 6	.096	.248	= .364	[-.289, .786]	[-.335, .773]
	ILF right	.045	.149	= .593	[-.400, .698]	[-.427, .811]
	IoFF left	.052	.162	= .611	[-.463, .787]	[-.476, .758]
	IoFF right	.064	-.047	= .870	[-.610, .516]	[-.648, .551]
	UF right	.057	.250	= .394	[-.326, .825]	[-.320, .897]
**AD**	AF left	.204	-.005	= .984	[-.509, .499]	[-.559, .527]
	AF right	.177	.091	= .799	[-.612, .793]	[-.674, .664]
	CB right	.094	.508	= .098	[-.093, 1.109]	[-.096, 1.150]
	CC 2	.194	-.251	= .427	[-.871, .369]	[-.935, .226]
	CC 3	.128	-.041	= .859	[-.500, .417]	[-.508, .407]
	CC 4	.133	.202	= .413	[-.282, .686]	[-.336, .714]
	CC 5	.170	.002	= .993	[-.462, .466]	[-.488, .460]
	CC 6	.204	-.104	= .668	[-.580, .372]	[-.609, .361]
	IoFF left	.177	-.278	= .428	[-.969, .412]	[-1.009, .298]
	SLF I left	.177	.080	= .778	[-.477, .636]	[-.520, .630]
	SLF I right	.166	.242	= .351	[-.268, .752]	[-.295, .820]
	SLF II left	.181	-.029	= .928	[-.658, .600]	[-.776, .492]
	SLF II right	.193	-.005	= .984	[-.528, .518]	[-.535, .538]
	SLF III right	.197	-.251	= .426	[-.872, .369]	[-.953, .244]

Note. MD, mean diffusivity; AD, axial diffusivity; AF, arcuate fasciculus; CB, cingulum bundle; CC, corpus callosum; ILF, inferior longitudinal fasciculus; IoFF, inferior occipito-frontal fasciculus; SLF, superior longitudinal fasciculus; UF, uncinate fasciculus; PTSD, post-traumatic stress disorder; mTBI, mild traumatic brain injury; HC3, heteroskedasticity-consistent; CI, confidence interval; BCa, bias-corrected accelerated. Linear models tested whether the association between childhood maltreatment severity and white matter microstructure varied jointly by PTSD and mTBI (CM × PTSD × mTBI). Reported are standardized three-way interaction coefficients (beta), model R^2, HC3 p-values with 95% CIs, and BCa bootstrap 95% CIs (5,000 resamples). All analyses were corrected for age and gender as covariates.

Childhood Maltreatment Subtypes

We performed additional post-hoc analyses to explore which type of CM subtype might have been driving the observed effects. For this purpose, we utilized the five subscales of the CTQ as independent variables: Emotional neglect, physical neglect, emotional abuse, physical abuse, and sexual abuse.

Across subscales, ~11-15% of participants reported moderate-to-severe maltreatment (**Table S9**). Emotional neglect (15%) and physical neglect (15%) were most frequently reported, followed by emotional abuse (14%), physical abuse (14%), and sexual abuse (11%) (**Table S10**). According to the criteria for moderate exposure by Bernstein et al. (2003), 25% of the participants experienced CM in at least one category. Notably, there was a high overlap among the different types of CM (**Figure S4**). Subscales were positively intercorrelated in expected directions (**Table S11**), consistent with prior reports that CTQ domains reflect related but distinct aspects of maltreatment.

Table S9. Prevalence of childhood maltreatment types (*N* = 299).

	None (%)	Low (%)	Moderate (%)	Severe (%)
**Emotional Neglect**	64.9	17.7	5.7	9.0
**Physical Neglect**	74.2	10.7	7.0	8.0
**Emotional Abuse**	74.2	11.4	5.4	9.0
**Physical Abuse**	70.9	15.4	4.0	9.7
**Sexual Abuse**	84.3	5.0	2.7	8.0

Note. Values are percentages of participants within each severity category using CTQ manual cutoffs (Bernstein & Fink, 1998; Bernstein et al., 2003).

Table S10. Summary of childhood maltreatment characteristics.

	N	Mean (SD)	Range	Cut-off	n	% of total sample
**CTQ total**	299	37.67 (17.69)	25 – 115	≥ 41	76	25%
**Emotional neglect**	299	9.14 (4.96)	5 – 25	≥ 13	44	15%
**Physical neglect**	299	6.94 (3.42)	5 – 21	≥ 10	45	15%
**Emotional abuse**	299	7.91 (4.50)	5 – 25	≥ 15	43	14%
**Physical abuse**	299	7.26 (3.94)	5 – 25	≥ 10	41	14%
**Sexual abuse**	299	6.41 (4.26)	5 – 25	≥ 8	32	11%

Note. CTQ, Childhood Trauma Questionnaire. This table displays frequencies of total childhood maltreatment and childhood maltreatment types according to the criteria for moderate exposure as outlined by Bernstein et al. (2003).

Table S11. Spearman correlations among CTQ subscales (*N* = 299).

	1. EN	2. PN	3. EA	4. PA	5. SA
**1. Emotional neglect**	—				
**2. Physical neglect**	0.64***	—			
**3. Emotional abuse**	0.70***	0.60***	—		
**4. Physical abuse**	0.49***	0.47***	0.60***	—	
**5. Sexual abuse**	0.41***	0.38***	0.47***	0.49***	—

Note. Values are Spearman's ρ. Lower triangle shown; diagonal entries are em dashes. EN = Emotional Neglect; PN = Physical Neglect; EA = Emotional Abuse; PA = Physical Abuse; SA = Sexual Abuse. ***p ≤ .001.

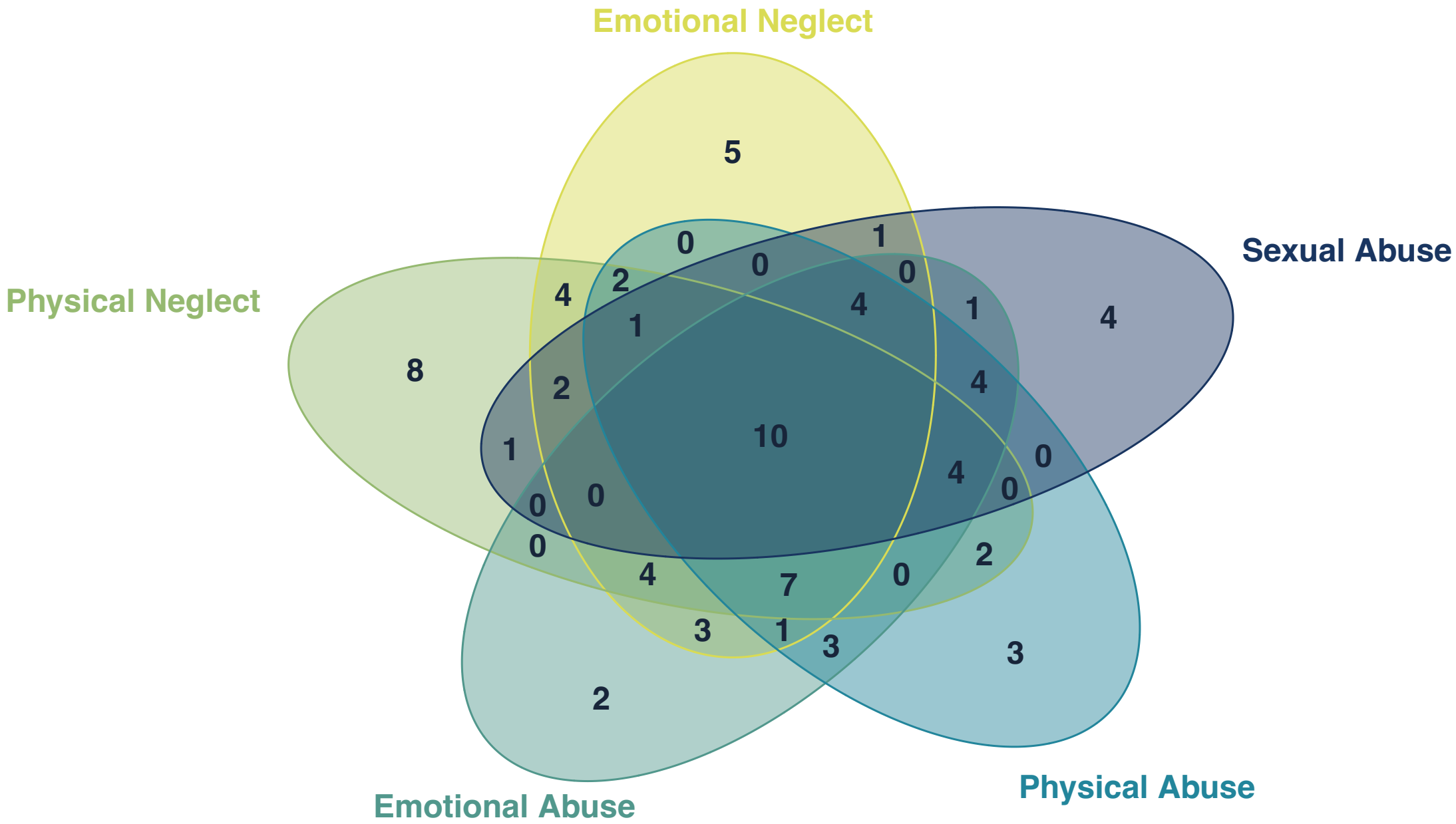

Figure S4. Childhood Maltreatment Types in the Sample.
As illustrated here, maltreatment exposure categories were largely overlapping. Numbers reflect number of participants reporting exposure to the indicated maltreatment types. The Childhood Trauma Questionnaire subscale classifications are defined by Bernstein and Fink (1998).

Associations between Childhood Maltreatment Subtypes and White Matter Microstructure

Supplementary analyses of the CM types revealed that predominantly emotional neglect and emotional abuse significantly contributed to the observed inverse associations between CM severity and MD and AD of various WM tracts. (**Figure S5** and **Table S12**).

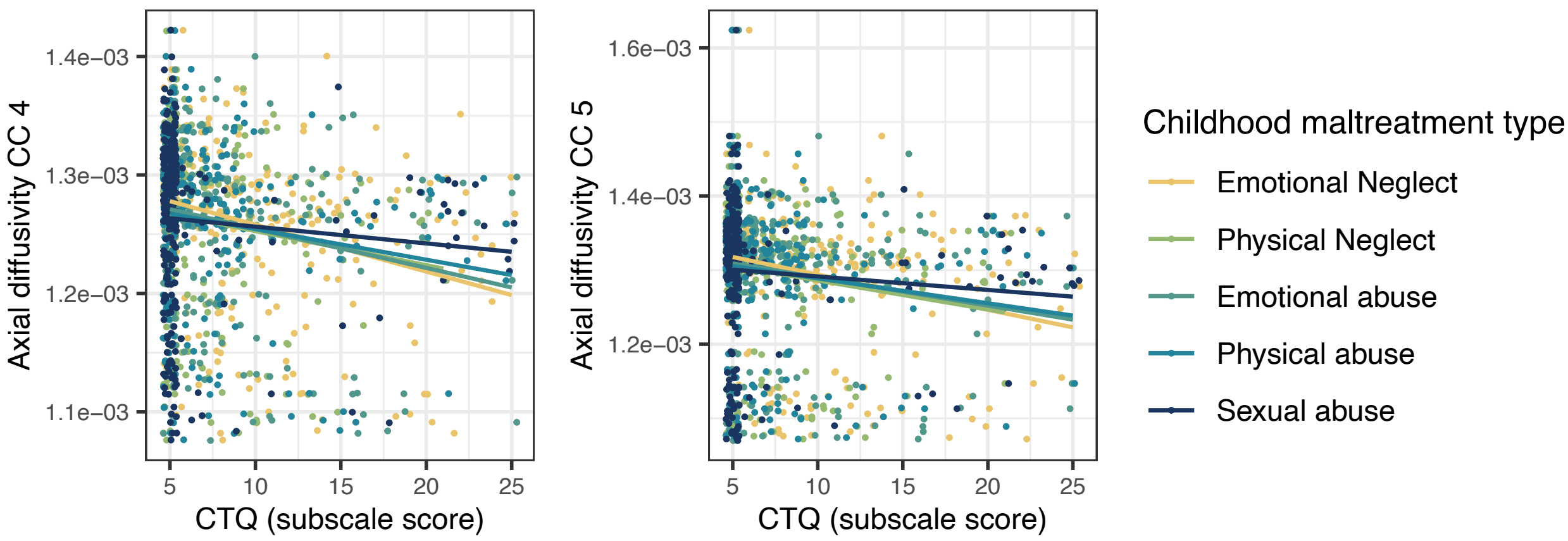

Figure S5. Associations between Childhood Maltreatment Subtypes and Axial Diffusivity in the Body Region of the Corpus Callosum.
Note. CC, corpus callosum; CTQ, Childhood Trauma Questionnaire. Only emotional neglect and emotional abuse were significantly negatively correlated with axial diffusivity in both CC4 and CC5.

Table S12. Multiple regression results using childhood maltreatment subtype as the criterion (*N* = 299).

CTQ subscale		Emotional neglect		Physical neglect		Emotional abuse		Physical abuse		Sexual abuse	
		p_{FDR}	beta.	p_{FDR}	beta.	p_{FDR}	beta.	p_{FDR}	beta.	p_{FDR}	beta.
**MD**	**AF right**	**.010**	**- .173**	.133	- .114	.037	- .143	.226	- .090	.163	- .101
	**CC 1**	**.025**	**- .153**	.080	- .129	**.005**	**- .185**	**.044**	**- .145**	.287	- .077
	**CC 2**	**.005**	**- .187**	.158	- .107	**.001**	**- .211**	.082	- .123	.151	- .105
	**CC 6**	**.001**	**- .210**	.178	- .101	**.002**	**- .199**	**.015**	**- .167**	.144	- .107
	**ILF right**	**.010**	**- .173**	.235	- .088	**.044**	**- .138**	.211	- .094	.212	- .091
	**IoFF left**	**.012**	**- .170**	**.040**	**- .150**	**.000**	**- .226**	.079	- .127	.079	- .127
	**Ioff right**	**.004**	**- .192**	.178	- .101	**.021**	**- .157**	.265	- .083	.265	- .083
	**UF right**	**.012**	**- .169**	.226	- .089	**.015**	**- .165**	.306	- .078	.287	- .078
**AD**	**AF left**	**.001**	**- .195**	.370	- .064	**.023**	**- .140**	.108	- .101	.592	- .038
	**AF right**	**.003**	**- .185**	.389	- .062	**.023**	**- .142**	.160	- .091	.528	- .046
	**CB right**	**.000**	**- .229**	.115	- .106	**.010**	**- .163**	.130	- .098	.418	- .060
	**CC 2**	**.029**	**- .137**	.367	- .066	**.019**	**- .149**	**.040**	**- .133**	.261	- .081
	**CC 3**	**.000**	**- .240**	**.044**	**- .132**	**.003**	**- .183**	**.007**	**- .167**	**.045**	**- .130**
	**CC 4**	**.000**	**- .246**	.131	- .102	**.007**	**- .169**	.087	- .111	.456	- .053
	**CC 5**	**.000**	**- .240**	.070	- .122	**.012**	**- .160**	.061	- .123	.408	- .063
	**CC 6**	**.015**	**- .155**	.347	- .072	**.020**	**- .149**	**.010**	**- .164**	.366	- .069
	**IoFF left**	**.028**	**- .139**	.362	- .069	**.030**	**- .138**	**.020**	**- .149**	.194	- .092
	**SLF I left**	**.000**	**- .221**	.099	- .109	**.016**	**- .150**	**.048**	**- .125**	.398	- .063
	**SLF I right**	**.000**	**- .217**	.104	- .107	.077	- .111	.186	- .084	.444	- .054
	**SLF II left**	**.009**	**- .160**	.173	- .091	**.042**	**- .125**	.088	- .108	.194	- .089
	**SLF II right**	**.000**	**- .226**	.099	- .107	**.017**	**- .146**	.087	- .107	.126	- .101
	**SLF III right**	**.010**	**- .158**	.416	- .057	**.039**	**- .129**	**.025**	**- .141**	.416	- .060
	**UF right**	**.000**	**- .248**	.061	- .127	**.001**	**- .204**	.051	- .128	**.032**	**- .141**

Note. CTQ, Childhood Trauma Questionnaire; FDR, false-discovery rate; MD, mean diffusivity; AD, axial diffusivity; CC, corpus callosum; AF, arcuate fasciculus; SLF, superior longitudinal fasciculus. Additional regression analyses using CTQ subscales as independent variables highlighted the substantial contributions of emotional neglect and emotional abuse to the observed effects. All analyses were corrected for age and gender.

Mediating Effect of White Matter Microstructure on the Association of Childhood Maltreatment Subtypes with PTSD and Post-concussive Symptom Severity

Additional statistical investigations found that predominantly emotional neglect contributed to the observed mediating effects of WM microstructure on the association of CM severity with PTSD (**Table S13**) and post-concussive symptom severity (**Table S14**).

Table S13. Mediation results of childhood maltreatment types on PTSD symptom severity (*N* = 299).

		Emotional neglect				Physical neglect				Emotional abuse				Physical abuse				Sexual abuse			
		Direct effect	Indirect effect			Direct effect	Indirect effect			Direct effect	Indirect effect			Direct effect	Indirect effect			Direct effect	Indirect effect		
		$b_{c'}$	b_{ab}	Perc. Bootstrap 95% CI		$b_{c'}$	b_{ab}	Perc. Bootstrap 95% CI		$b_{c'}$	b_{ab}	Perc. Bootstrap 95% CI		$b_{c'}$	b_{ab}	Perc. Bootstrap 95% CI		$b_{c'}$	b_{ab}	Perc. Bootstrap 95% CI	
				Lower	Upper			Lower	Upper			Lower	Upper			Lower	Upper			Lower	Upper
MD	CC 6	.351***	.092	**.006**	**.219**	.411***	.078	-.007	.223	.334***	.101	**.010**	**.244**	.411***	.078	-.007	.223	.295***	.071	- .002	.183
AD	AF left	.349***	.098	**.012**	**.224**	.415***	.060	-.029	.204	.340***	.079	-.002	.200	.415***	.060	-.029	.204	.305***	.031	- .042	.127
	CC 2	.353***	.084	**.008**	**.181**	.414***	.065	-.049	.189	.334***	.104	-.001	.228	.414***	.065	-.049	.189	.296***	.065	- .030	.167
	CC 3	.353***	.082	**.004**	**.191**	.410***	.085	**.012**	**.200**	.337***	.082	**.012**	**.208**	.410***	.085	**.012**	**.200**	.294***	.076	**.013**	**.165**
	CC 4	.344***	.117	**.017**	**.248**	.409***	.092	**.004**	**.234**	.337***	.088	**.008**	**.216**	.409***	.092	**.004**	**.234**	.301***	.044	- .022	.138
	CC 5	.331***	.161	**.056**	**.301**	.400***	.137	**.017**	**.307**	.329***	.123	**.013**	**.275**	.400***	.137	**.017**	**.307**	.296***	.064	- .022	.174
	CC 6	.342***	.121	**.027**	**.237**	.409***	.090	-.049	.252	.321***	.161	**.045**	**.318**	.409***	.090	-.049	.252	.294***	.072	- .030	.197
	SLF I left	.356***	.150	**.051**	**.278**	.389***	.142	**.016**	**.306**	.280***	.125	**.016**	**.273**	.389***	.142	**.016**	**.306**	.252***	.075	- .024	.202
	SLF I right	.343***	.117	**.025**	**.237**	.407***	.100	**.002**	**.245**	.341***	.075	-.005	.195	.407***	.100	**.002**	**.245**	.301***	.047	- .028	.153
	SLF II left	.345***	.116	**.017**	**.242**	.407***	.101	-.014	.268	.334***	.104	-.010	.248	.407***	.101	-.014	.268	.292***	.083	- .017	.199
	SLF II right	.346***	.107	**.013**	**.233**	.408***	.093	**.004**	**.234**	.338***	.084	**.000**	**.205**	.408***	.093	**.004**	**.234**	.293***	.077	**.010**	**.173**

Note. Total effects: $b_{c,\ Emotional\ neglect}$ = .377, t(299) = 7.015, *p* <.001; $b_{c,\ Physical\ neglect}$ = .427, t(299) = 8.244, *p* <.001; $b_{c,\ Emotional\ abuse}$ = .355, t(299) = 7.149, p <.001; $b_{c,\ Physical\ abuse}$ = .358, t(299) = 6.713, p <.001; $b_{c,\ Sexual\ abuse}$ = .312, t(299) = 5.776, *p* <.001. Additional mediation analyses using childhood maltreatment types as independent variables highlighted the substantial contributions of emotional neglect and emotional abuse to the observed effects. All analyses were corrected for age and gender. ***p ≤ .001.

Table S14. Mediation results of childhood maltreatment types on post-concussive symptom severity (*N* = 177).

		Emotional neglect				Physical neglect				Emotional abuse				Physical abuse				Sexual abuse			
		Direct effect	Indirect effect			Direct effect	Indirect effect			Direct effect	Indirect effect			Direct effect	Indirect effect			Direct effect	Indirect effect		
		$b_{c'}$	b_{ab}	Perc. Bootstrap 95% CI		$b_{c'}$	b_{ab}	Perc. Bootstrap 95% CI		$b_{c'}$	b_{ab}	Perc. Bootstrap 95% CI		$b_{c'}$	b_{ab}	Perc. Bootstrap 95% CI		$b_{c'}$	b_{ab}	Perc. Bootstrap 95% CI	
				Lower	Upper			Lower	Upper			Lower	Upper			Lower	Upper			Lower	Upper
MD	CC 2	.343***	.104	**.017**	**.271**	.329***	.101	-.049	.300	.315***	.128	**.019**	**.309**	.312***	.124	**.011**	**.355**	.265***	.080	-.013	.231
	CC 6	.316***	.182	**.058**	**.350**	.326***	.113	-.058	.331	.302***	.168	**.025**	**.360**	.300***	.168	**.015**	**.396**	.278***	.040	-.110	.201
AD	AF left	.338***	.118	**.017**	**.263**	.336***	.070	-.053	.255	.321***	.111	**.012**	**.257**	.316***	.108	**.000**	**.277**	.286***	.013	-.079	.122
	AF right	.330***	.140	**.033**	**.285**	.331***	.095	-.052	.297	.314***	.130	**.019**	**.282**	.313***	.118	-.018	.293	.285***	.017	-.102	.139
	CB right	.314***	.186	**.069**	**.349**	.328***	.106	-.069	.329	.305***	.160	**.032**	**.338**	.304***	.151	**.002**	**.371**	.271***	.060	-.062	.220
	CC 4	.301***	.223	**.087**	**.410**	.318***	.146	-.023	.386	.294***	.194	**.054**	**.395**	.292***	.197	**.041**	**.436**	.275***	.048	-.078	.186
	CC 5	.323***	.162	**.043**	**.328**	.324***	.122	-.037	.341	.316***	.123	**.005**	**.292**	.312***	.121	-.027	.330	.286***	.013	-.100	.138
	IoFF right	.343***	.103	**.007**	**.244**	.336***	.072	-.045	.253	.322***	.106	**.007**	**.248**	.317***	.105	**.004**	**.265**	.284***	.020	-.069	.110
	SLF I left	.305***	.213	**.079**	**.385**	.321***	.136	-.046	.387	.300***	.175	**.045**	**.358**	.288***	.212	**.060**	**.446**	.269***	.067	-.048	.217
	SLF I right	.321***	.168	**.062**	**.303**	.332***	.087	-.064	.278	.321***	.110	-.002	249	.314***	.117	-.006	.298	.280***	.034	-.080	.169
	SLF II left	.336***	.124	**.017**	**.282**	.325***	.119	-.019	.333	.317***	.122	**.012**	**.283**	.311***	.128	**.003**	**.325**	.271***	.062	-.025	.179
	SLF II right	.337***	.122	**.009**	**.273**	.325***	.119	**.000**	**.312**	.317***	.123	**.016**	**.265**	.311***	.125	**.016**	**.288**	.268***	.072	-.007	.181

Note. Total effects: $b_{c,\ Emotional\ neglect}$ = .379, t(177) = 5.428, *p* <.001; $b_{c,\ Physical\ neglect}$ = .353, t(177) = 4.977, *p* <.001; $b_{c,\ Emotional\ abuse}$ = .356, t(177) = 4.999, p <.001; $b_{c,\ Physical\ abuse}$ = .345, t(177) = 4.894, p <.001; $b_{c,\ Sexual\ abuse}$ = .290, t(177) = 4.036, *p* <.001. Additional mediation analyses using childhood maltreatment types as independent variables highlighted the substantial contributions of emotional neglect and emotional abuse to the observed effects. All analyses were corrected for age and gender. ***p ≤ .001.
